# The Tissue-Specific RNA Binding Protein T-STAR Controls Regional Splicing Patterns of *Neurexin* Pre-mRNAs in the Brain

**DOI:** 10.1371/journal.pgen.1003474

**Published:** 2013-04-25

**Authors:** Ingrid Ehrmann, Caroline Dalgliesh, Yilei Liu, Marina Danilenko, Moira Crosier, Lynn Overman, Helen M. Arthur, Susan Lindsay, Gavin J. Clowry, Julian P. Venables, Philippe Fort, David J. Elliott

**Affiliations:** 1 Institute of Genetic Medicine, Newcastle University, Newcastle upon Tyne, United Kingdom; 2 Institute of Neuroscience, Newcastle University, Newcastle upon Tyne, United Kingdom; 3 Universités Montpellier 2 et 1, UMR 5237, Centre de Recherche de Biochimie Macromoléculaire, CNRS, Montpellier, France; Medical Research Council Human Genetics Unit, United Kingdom

## Abstract

The RNA binding protein T-STAR was created following a gene triplication 520–610 million years ago, which also produced its two parologs Sam68 and SLM-1. Here we have created a T-STAR null mouse to identify the endogenous functions of this RNA binding protein. Mice null for T-STAR developed normally and were fertile, surprisingly, given the high expression of T-STAR in the testis and the brain, and the known infertility and pleiotropic defects of Sam68 null mice. Using a transcriptome-wide search for splicing targets in the adult brain, we identified T-STAR protein as a potent splicing repressor of the alternatively spliced segment 4 (AS4) exons from each of the *Neurexin1-3* genes, and exon 23 of the *Stxbp5l* gene. T-STAR protein was most highly concentrated in forebrain-derived structures like the hippocampus, which also showed maximal *Neurexin1-3* AS4 splicing repression. In the absence of endogenous T-STAR protein, *Nrxn1-3* AS4 splicing repression dramatically decreased, despite physiological co-expression of Sam68. In transfected cells *Neurexin3* AS4 alternative splicing was regulated by either T-STAR or Sam68 proteins. In contrast, *Neurexin2* AS4 splicing was only regulated by T-STAR, through a UWAA-rich response element immediately downstream of the regulated exon conserved since the radiation of bony vertebrates. The AS4 exons in the *Nrxn1* and *Nrxn3* genes were also associated with distinct patterns of conserved UWAA repeats. Consistent with an ancient mechanism of splicing control, human T-STAR protein was able to repress splicing inclusion of the zebrafish *Nrxn3* AS4 exon. Although *Neurexin1-3* and *Stxbp5l* encode critical synaptic proteins, T-STAR null mice had no detectable spatial memory deficits, despite an almost complete absence of AS4 splicing repression in the hippocampus. Our work identifies T-STAR as an ancient and potent tissue-specific splicing regulator that uses a concentration-dependent mechanism to co-ordinately regulate regional splicing patterns of the *Neurexin1-3* AS4 exons in the mouse brain.

## Author Summary

Alternative splicing plays a key role in animal development and is largely controlled by the expression of RNA binding proteins. Most RNA binding proteins exist as families of sister proteins called paralogs, which result from gene amplification, including T-STAR, which is closely related to Sam68 and SLM-1. T-STAR, Sam68, and SLM-1 usually behave identically in splicing control in transfected cells. Here we report the physiological functions of T-STAR protein by knocking its parent gene out in the mouse. Surprisingly we observed no defects in germ cell maturation without T-STAR protein, an unexpected result given T-STAR protein is mainly expressed in the testis and its paralog Sam68 is essential for male fertility. Instead, we find T-STAR controls a panel of splicing targets that encode important synaptic proteins. T-STAR acts as a potent splicing repressor to establish regional splicing patterns of these target exons in the brain. Forebrain-derived structures like the hippocampus strongly express T-STAR protein to repress these target exons. Some T-STAR regulated splicing targets overlap with Sam68, but T-STAR also regulates its own distinct targets. Comparative genomic analyses are consistent with an ancient mechanism of splicing control by T-STAR that has been conserved since the radiation of bony vertebrates.

## Introduction

RNA binding proteins expand the functional complexity of the transcriptome by specifying which exons are spliced into mRNAs at key developmental steps, and make a significant contribution to animal development and complexity [1]–[7]. Splicing takes place in the spliceosome, which consists of 5 snRNAs and up to 200 proteins including a core of essential components and many facultative proteins peripheral to the core [8]. Among the latter are a group of alternative splicing factors whose presence is limiting for regulation of specific subsets of alternative exons. Intriguingly, most alternative splicing factors occur as families of paralogs including Sam68, T-STAR and SLM-1; TRA2α and Tra2β; PTBP1, 2 and 3; MBNL1, 2 and 3; RBFOX1, 2 and 3; TIAL and TIA-1; and hnRNPG and hnRNPG-T amongst others [9]. In some cases splicing regulator paralogs have been shown to have important and functionally distinct roles within animals [10]–[15]. However the existence of multiple forms of these splicing factors poses a conundrum as to whether their existence simply enables each family to have complex spatiotemporal expression patterns, or whether the individual members of each family might have distinct RNA targets.

Here we address the function of T-STAR protein, one of the three homologous KHDRBS splicing regulator proteins. Three *KHDRBS* genes encode T-STAR, Sam68 and SLM-1 proteins (encoded by the *KHDRBS3*, *KHDRBS1* and *KHDRBS2* genes respectively), and evolved around the same time by a triplication of a common ancestral gene between the divergence of hyperoartia and jawed fish around 520 to 610 million years ago (Figure S1). Each of these KHDRBS proteins contain a STAR domain (comprising a ‘KH’-type RNA binding domain flanked by a pair of conserved sequences called QUA1 and QUA2 domains) which is involved in both RNA processing and protein interactions, and a number of other protein domains implicated in cellular signalling pathways (notably SH3 binding and WWW motifs, as well as conserved tyrosines which contribute to candidate SH2 binding domains) [16]–[19]. Each of the mammalian KHDRBS proteins have different but overlapping anatomic expression patterns [20]–[23]. T-STAR protein (also known as SLM-2) is primarily expressed in the testis and the brain [22]. Sam68 protein is expressed ubiquitously, while rat SLM-1 is expressed in the brain with more limited expression in the testis [20]. Sam68 protein becomes functionally sequestered by a triplet repeat sequence in the neurological disease Fragile X Tremor Associated Ataxia Syndrome (FXTAS), and T-STAR is sometimes amplified in medulloblastoma [24], [25].

The only member of the KHDRBS protein family to have been investigated genetically in vertebrates is Sam68. Experiments done with Sam68 knockout mice have shown that Sam68 has important functions in development and physiology [26]–[32]. Sam68 protein is essential for male germ cell development, even though it is co-expressed with T-STAR in the testis. Sam68 null mice are infertile as a result of defects in translational control of stored mRNAs during spermatogenesis [29]–[31]. Sam68 null mice also have behavioural deficits and poor motor control [28]. Sam68 regulates splicing control of several exons during neuronal differentiation *in vitro* [33], and regulates signal-dependent splicing of *Neurexin1* (abbreviated *Nrxn1*) mRNA isoforms in the cerebellum *in vivo* [27]. The neurexins are amongst the most diverse protein types in the body although they are encoded by just three genes (*Nrxn1-3*). This molecular diversity is generated by variable inclusion of five alternatively spliced regions into the *Nrxn1-3* mRNAs and by use of two alternative promoters, to produce thousands of different mRNA and protein products [34]–[36]. Splicing inclusion of alternatively spliced segment 4 (abbreviated AS4) has been proposed to regulate neurexin protein-protein interactions, guide the formation of synapses [27], [37], [38], and to comprise an important part of a code which establishes how neurons connect with their ligands and how synapses assemble [39]–[41].

T-STAR and Sam68 have very similar activities in transfected cells, e.g. both regulate splicing control of a cassette exon in the rat *CD44* gene [21], [22]. A key question is why two apparently very similar proteins like T-STAR and Sam68 have both been maintained in evolution? Here we have addressed the physiological functions of T-STAR protein by creating a null *Khdrbs3* allele and analysing the resulting mice. Surprisingly given its high expression level in the testis, we find that T-STAR is not essential for germ cell development. Instead we find that T-STAR is in fact the critical protein which establishes the exquisite regional splicing patterns of the *Neurexin* AS4 exon in the brain.

## Results

### The *Khdrbs3* gene is not essential for mouse development or male fertility

**Figure 1:**
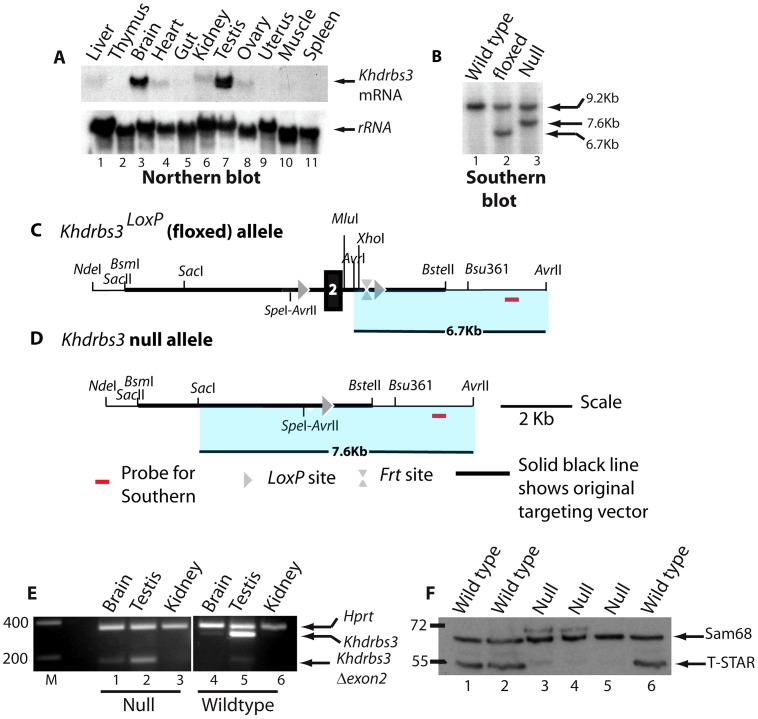
Generation of a null *Khdrbs3* allele. (A) Northern analysis of different adult mouse tissues to detect expression of *Khdrbs3* (upper panel) and small subunit rRNA (lower panel). (B) The genomic structure of the *Khdrbs3* alleles from wild type, floxed, and null mice mice were monitored using Southern blotting and the probe indicated in parts C–D. The Southern blot demonstrates that the cross with a PGK-Cre mouse successfully removed exon 2 from the genomic DNA. (C) Genomic structure of the *Khdrbs3^LoxP^* conditional allele in which exon 2 of the *Khdrbs3* gene is flanked by *Lox*P sites. (D) Genomic structure of the null (*Khdrbs3^−^*) allele from which exon 2 has been deleted by Cre-mediated recombination. (E) Multiplex RT-PCR analysis of *Khdrbs3* and *Hprt* mRNA levels in different mouse tissues. The size markers are shown in nucleotides. (F) Western blot analysis of Sam68 and T-STAR protein levels in the testes of wild type and *Khdrbs3* null mice using an antibody that recognizes T-STAR and Sam68. The position of the size markers are shown in KDa.

High levels of *Khdrbs3* mRNA were detected in the mouse testis by Northern blotting (Figure 1A). We therefore hypothesized that T-STAR protein would have an essential role in male germ cell development. To test this prediction we used standard techniques to create a null allele of the *Khdrbs3* gene (Figure 1B–1D). Briefly, we created a conditional allele in which exon 2 of the mouse *Khdrbs3* gene was flanked by *LoxP* sites, and then deleted this exon by expression of Cre-recombinase under control of the ubiquitous *PGK* promoter (Figure 1B–1D. All experimental details are provided in the Methods section).

**Figure 2:**
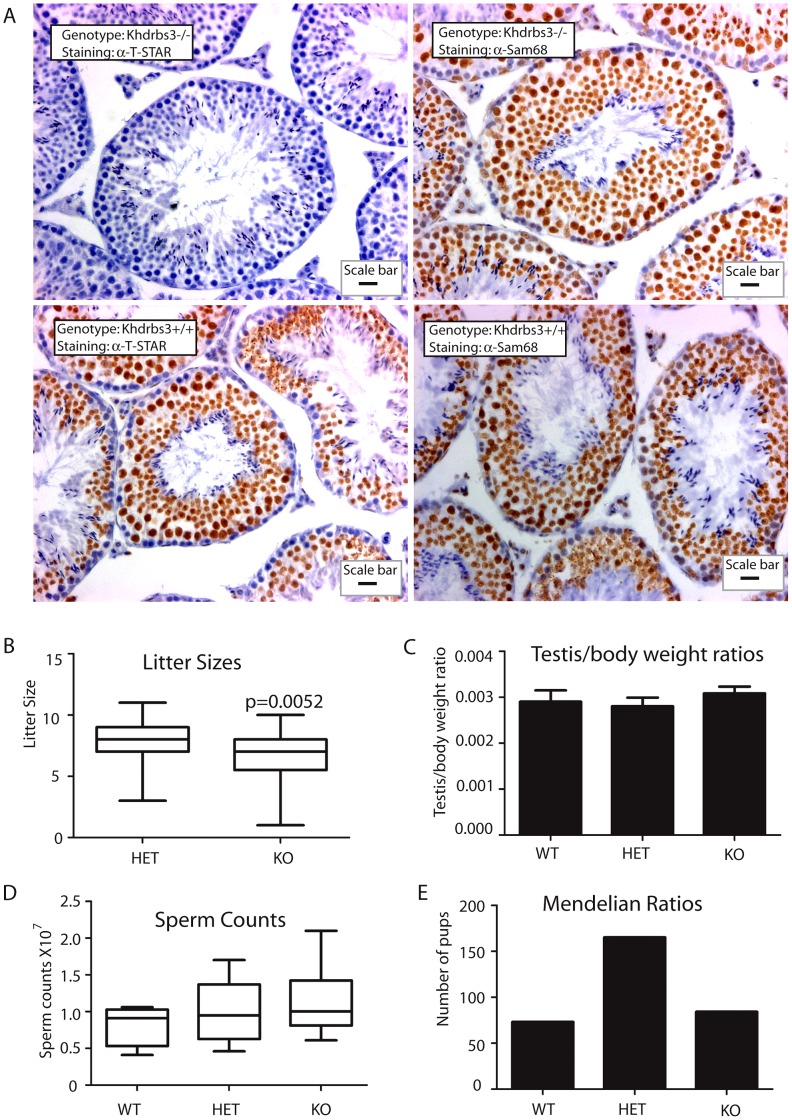
T-STAR is not required for male fertility in the mouse. (A) Histology of testis from wild type and *Khdrbs3^−/−^* animals. Paraffin-embedded sections were probed for T-STAR using a T-STAR specific antibody or Sam68 using a Sam68 specific antibody and counterstained using haematoxylin. The scale bar is equivalent to 20 µm. (B) Litter sizes obtained for *HET* (n = 40 litters) and *Khdrbs3^−/−^* (n = 23 litters) mice. Slightly smaller than average litter sizes were observed for the KO mice. (C) Testis-body weight ratios were not significantly different between WT (*Khdrbs3^+/+^*), HET and KO mice indicating no significant defect in adult testis development in the absence of T-STAR protein on a C57BL6 background or a mixed C57Bl6-129 background. (D) Sperm counts of wild type (n = 4); HET (n = 10) and KO (n = 9) mice indicate no reduction in sperm count associated with the knockout allele. (E) Mendelian ratios of litters born from breeding HET mice correspond to the expected 1∶2∶1 ratio, and so indicate no lethality associated with the KO allele (n = 40 litters).

To confirm that our strategy had been successful in generating a null allele of *Khdrbs3*, protein and RNA expression levels were analysed in the different genotype mice. Multiplex RT-PCR analysis using primers specific to *Khdrbs3* exons 1 and 3 detected high levels of the *Khdrbs3* mRNA relative to *Hprt* in wild type testis and lower levels in the brain (Figure 1E, lanes 4–6). Targeted deletion of exon 2, which is 119 nucleotides long, introduces a frameshift into the *Khdrbs3* mRNA resulting in early truncation of the open reading frame. A short RT-PCR product (corresponding to exon 2 deletion) was exclusively detected in RNA isolated from *Khdrbs3^−/−^* mice (Figure 1E, lanes 1–3). The frameshift caused by exon 2 deletion likely induces mRNA instability through nonsense mediated decay (NMD), since much lower levels of this exon 2 skipped version of *Khdrbs3* mRNA were detected in *Khdrbs3^−/−^* mouse testis compared with mRNA levels in the wild type genotype. Only Sam68 and not T-STAR protein was detected in the *Khdrbs3^−/−^* mice by Western analysis (Figure 1F) and immunohistochemistry (Figure 2A), although both T-STAR and Sam68 were detected in the testes of wild type mice. Hence we conclude exon 2 deletion from the *Khdrbs3* gene creates a true T-STAR knockout allele.

Male germ cell development proceeded normally in the absence of T-STAR protein. Seminiferous tubule morphology from the T-STAR knockout testis was indistinguishable from wild type (Figure 2A). Male T-STAR knockout mice were also fertile. Within our sample population, T-STAR knockout males sired slightly smaller litters compared with heterozygous males (unpaired t test, P = 0.0052; Mann Whitney test, P = 0.0067) (Figure 2B). However, average adult testis/body weight ratios were not significantly different in each of the three genotypes (wild type, knockout and heterozygote) indicating no significant issues with adult testis development (Figure 2C). Also there was no significant reduction in epididymal sperm number (Figure 2D), nor increase in abnormal sperm morphology in the *Khdrbs3^−/−^* mice (data not shown). Sperm from wild type and *Khdrbs3^−/−^* mice were also equally able to undergo the acrosome reaction (data not shown) indicating no problems with fertilisation.

Normal Mendelian ratios of each genotype were obtained after heterozygous crosses (Figure 2E). Hence there was no embryonic lethality or wave of perinatal mortality in mice without the *Khdrbs3* gene, unlike those reported for the Sam68 null mice [31]. Mice containing the T-STAR knockout alleles were bred onto C57/Bl6 and 129 backgrounds. On both genetic backgrounds *Khdrbs3^−/−^* knockout mice were apparently healthy, so we concentrated our subsequent analysis on the C57/Bl6 background.

### T-STAR protein regulates splicing inclusion of the *Neurexin1-3* AS4 exons in the mouse brain

The above data unexpectedly showed that T-STAR protein is not required for male germ cell development. We therefore set out to identify defects in the brain which is the other major site of T-STAR protein expression [22]. We purified RNA from wild type and *Khdrbs3^−/−^* mouse brain and carried out a transcriptome-wide search for alternative splicing differences using a medium throughput PCR platform [42]. The resulting data was subjected to quality control (see Methods) and plotted to show the levels of percentage splicing inclusion in wild type brain against the corresponding value in the knockout brain (Figure S2 and Dataset S1). We then independently analysed splice isoform ratios in multiple replicates of wild type, heterozygote and knockout mice. From these we confirmed four strong and robust splicing differences reproducible between individual mice (n = 3 of each genotype).

**Figure 3:**
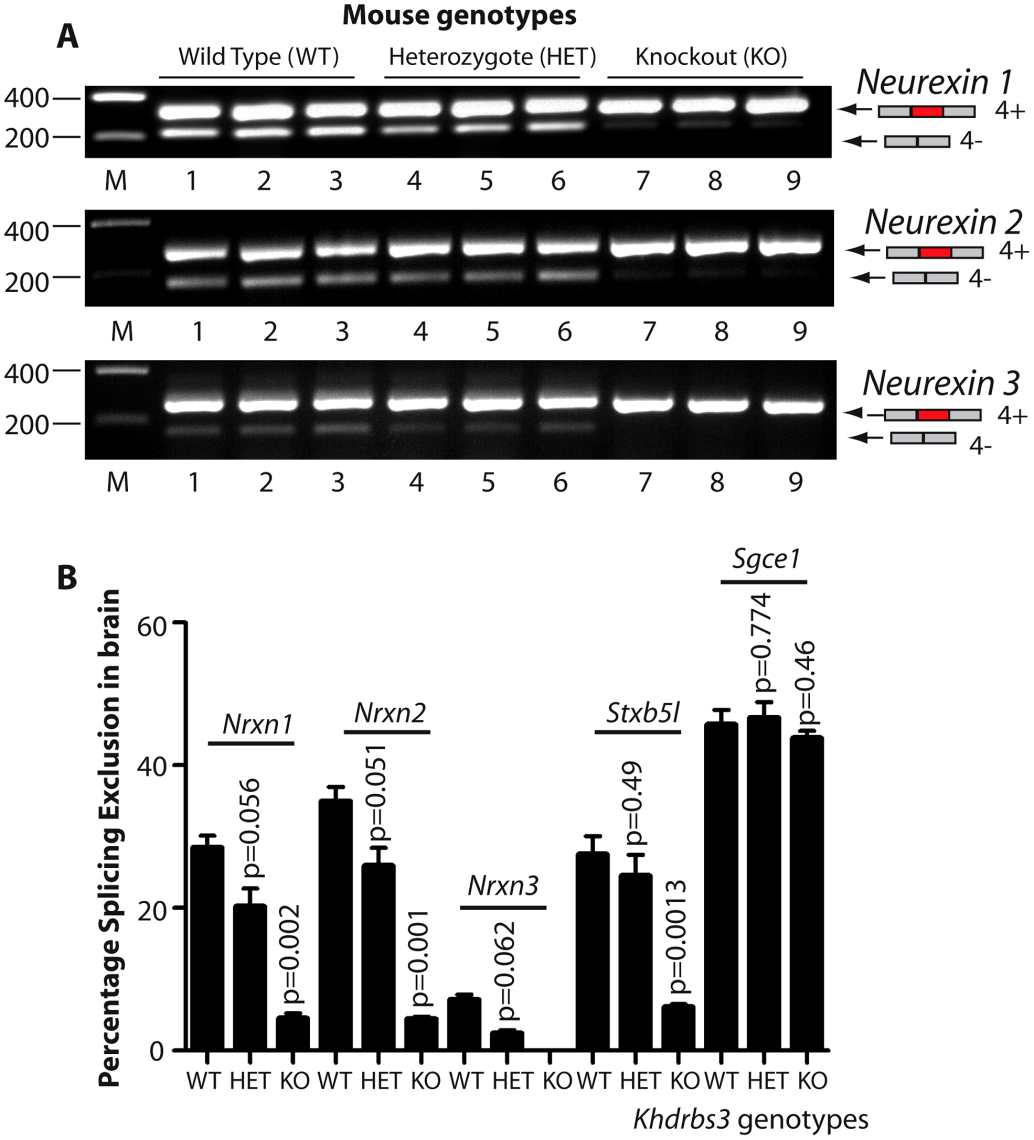
T-STAR protein is a dose-dependent splicing regulator of *Nrxn1-3* AS4 in the mouse brain. (A) Agarose gels showing levels of AS4 splicing inclusion from each of the *Nrxn1*, *Nrxn2* and *Nrxn3* genes using three different mice from each genotype. (B) Percentage Splicing Exclusion levels of *Nrxn1-3* AS4 and *Stxbp5l* exon 23 measured measured by RT-PCR and capillary gel electrophoresis in wild type (WT), *Khdrbs3^+/−^* (HET) and *Khdrbs3^−/−^* mice (KO) (n = 3 mice for each genotype). The p values were calculated from two tailed unpaired t tests, and error bars represent standard errors of the mean.

The four identified T-STAR regulated mRNA splice isoforms in the adult brain were the *Neurexin1-3* variants which differ as to whether they include exon 20 (hereafter referred to as alternatively spliced segment 4, or AS4) and *Stxbp5l* exon 23 (Figure 3A–3B). In each case higher levels of exon skipping were observed in the wild type brain compared to the *Khdrbs3^+/−^* heterozygote, and almost complete AS4 exon inclusion in the *Khdrbs3^−/−^* (T-STAR knockout) mouse brain. Differences in splicing exclusion levels of the wild type and T-STAR knockout mouse brains were statistically significant (Figure 3B, n = 3 mice). These data show T-STAR operates as a splicing repressor of these exons. In contrast, no significant splicing changes between wild type and T-STAR knockout mice were seen in splice isoforms made from the known Sam68 target exon *Sgce1* [29], [33] (Figure 3B).

### T-STAR protein expression establishes regional splicing regulation of *Nrxn1-3* exon AS4 in the mouse brain

**Figure 4:**
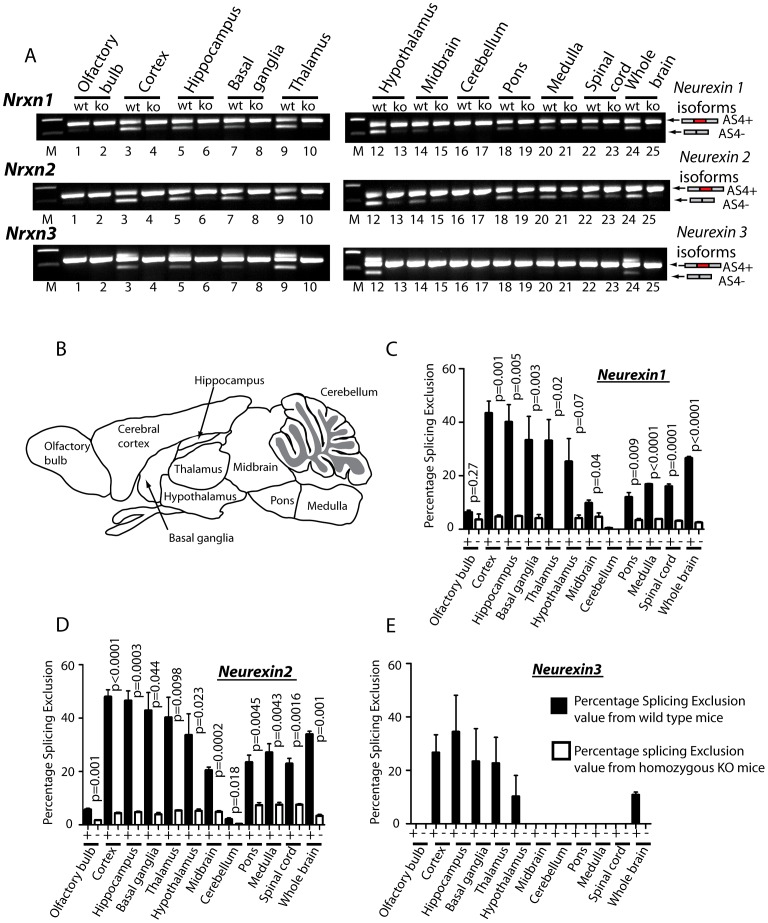
T-STAR protein regulates region-specific splicing of *Neurexin1-3 AS4* in the mouse brain. (A) *Neurexin* splicing regulation in different regions of the mouse brain (B) Schematic of the different mouse brain regions used for analysis. (C–E) Percentage splicing exclusion in different regions of the mouse brain (n = 3 mice from each genotype) measured in RNA samples from wild type (column +) and knockout (column −) mice for AS4 of (C) *Nrxn1*, (D) *Nrxn2* and (E) *Nrxn3*. The error bars correspond to the standard error of the mean. Statistical significances were calculated using a two tailed unpaired t test. No splicing exclusion was observed for *Nrxn3* in the absence of T-STAR protein in any brain region.

Splicing of *Nrxn1-3* AS4 exons are regionally controlled in the adult mouse brain (Figure 4A–4B), with high levels of skipping in forebrain-derived structures like the cortex and hippocampus, and much lower levels of skipping in hindbrain structures like the cerebellum [27]. RT-PCR analysis of different brain regions showed these regional splicing patterns were totally abolished in the brains of T-STAR null mice (Figure 4A shows results from an individual wild type and a knock out mouse, and Figure 4C–4E show quantitative data from 3 brains of each genotype). As a result of T-STAR deletion, splicing repression levels of *Nrxn1-3* AS4 were similar across each adult brain region from the knockout mice. For example, in the thalamus levels of percentage splicing exclusion for *Nrxn1-3* AS4 dropped from ∼50% in the wild type mouse to ∼0–5% in the *Khdrbs3^−/−^* mouse.

Although maximal AS4 splicing repression took place in forebrain-derived regions in wild type mice, there was also reduced but detectable *Nrxn1* and *Nrxn2* AS4 repression in the olfactory bulb, midbrain and cerebellum, and intermediate levels of AS4 repression in the pons, medulla and spinal cord. In each of these brain regions we also observed significantly reduced *Nrxn1* and *Nrxn2* AS4 splicing exclusion in the knockout (*Khdrbs3^−/−^*) genetic background compared to wild type (Figure 4C–4D). We conclude that T-STAR protein affects *Nrxn1-3* AS4 splicing patterns across the whole adult brain, but has a very substantial effect in forebrain-derived structures which normally show maximal splicing repression of this exon.

**Figure 5:**
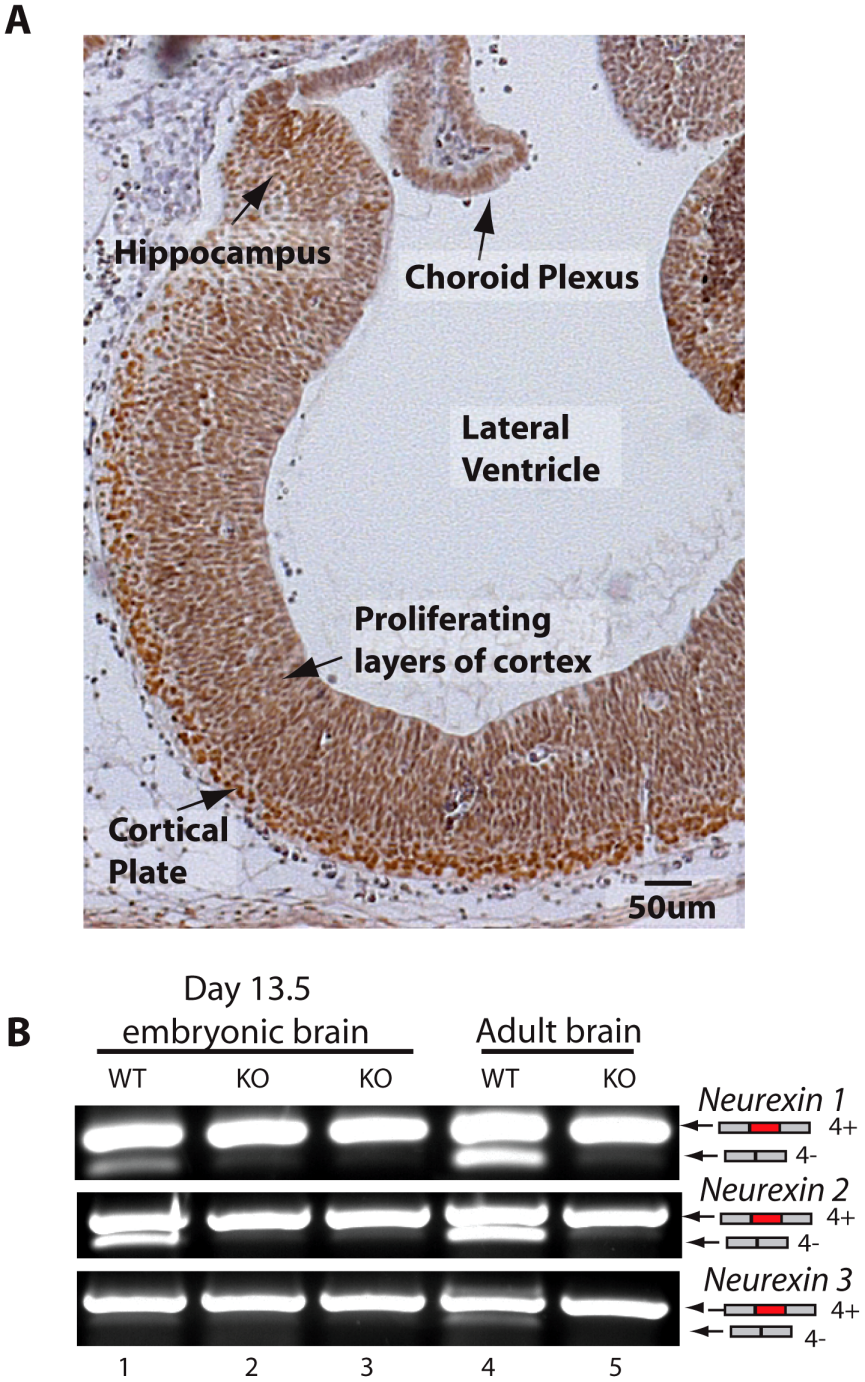
T-STAR protein is expressed in the embryonic brain. (A) Section of embryonic brain (13.5 day embryo) including most of the forebrain region, stained for T-STAR (brown) and counterstained with haematoxylin (blue). (B) *Nrxn1-3* exon AS4 splicing patterns in wild type and T-STAR knockout 13.5d embryonic brain.

The above experiments were carried out in the adult brain, but we also observed strong expression of T-STAR in the embryonic brain (embryonic day 13.5, Figure 5A). Embryonic T-STAR protein expression was particularly strong in the cortical plate, but less in the proliferating layers of the embryonic cortex. Strong embryonic T-STAR expression was also detected in the embryonic hippocampus, and in the epidermal layer of the choroid plexus. At this same stage of embryonic brain development the *Neurexin* AS4 exons also showed splicing exclusion, which was blocked in the T-STAR null mouse (Figure 5B).

### Regional protein concentrations and AS4 splicing patterns suggest T-STAR operates as a concentration-dependent splicing switch

**Figure 6:**
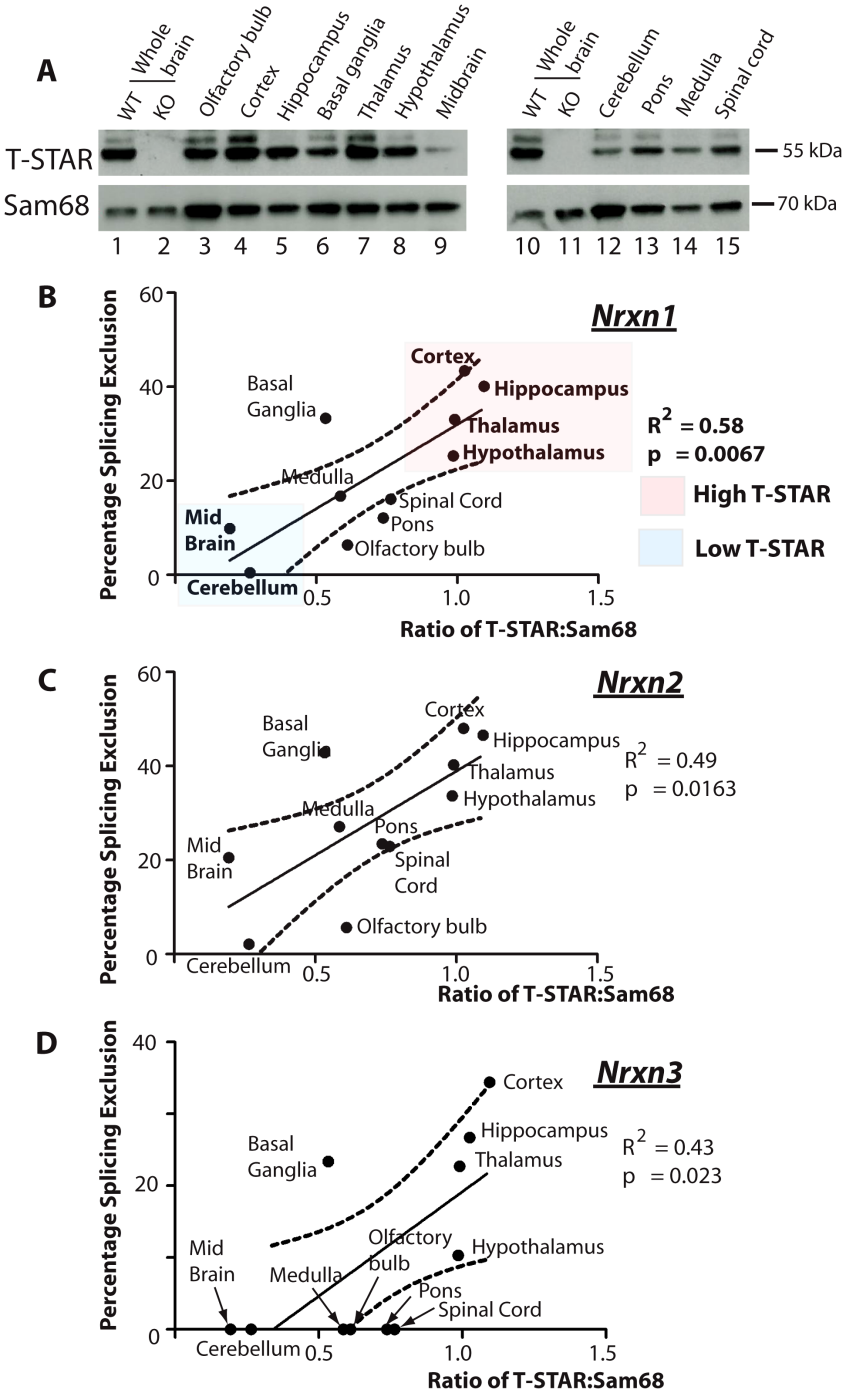
T-STAR protein concentration correlates with *Nrxn1-3* AS4 alternative splicing patterns. (A) Expression levels of T-STAR and Sam68 protein in different regions of the mouse brain were measured using Western blotting. The same filters were first probed with antisera specific for T-STAR, and then stripped and reprobed with an antisera specific for Sam68. (B–D) Levels of *Nrxn1-3* AS4 Percentage Splicing Exclusion in each brain region plotted against the ratio of T-STAR: Sam68 protein quantified from the Western blot shown in (A). The dashed line is the 95% confidence limit of the best fit line.

To establish how T-STAR might function as a regional *Nrxn* AS4 splicing regulator, we next monitored regional T-STAR and Sam68 protein expression in the adult mouse brain using Western blots (Figure 6A). T-STAR protein migrated as a major isoform of ∼55 kDa, with a minor protein isoform migrating with a slightly larger molecular weight. This minor T-STAR protein isoform was particularly enriched in the cortex, and has not been further investigated here. Both T-STAR protein isoforms disappeared in the T-STAR null background, whilst levels of Sam68 protein were unaffected.

We plotted the observed levels of splicing repression for *Nrxn1-3* AS4 exons in each brain region against the ratio of major T-STAR protein isoform expression relative to Sam68. Linear regression analysis indicated a positive and statistically significant correlation in each case (Figure 6B–6D). Higher ratios of T-STAR:Sam68 protein expression were found in forebrain-derived structures, which also had maximal *Nrxn1-3* exon AS4 skipping (the cortex, hippocampus, basal ganglia, thalamus and hypothalamus). Lowest ratios of T-STAR:Sam68 protein expression were detected in the olfactory bulb and the cerebellum which also showed lowest levels of *Nrxn1-3* AS4 alternative splicing regulation. These data are consistent with an AS4 splicing switch mechanism driven by regional concentrations of T-STAR protein in the adult brain.

### *Nrxn1-3* AS4 splicing regulation depends on endogenous expression of T-STAR protein even when there is physiological co-expression of Sam68

**Figure 7:**
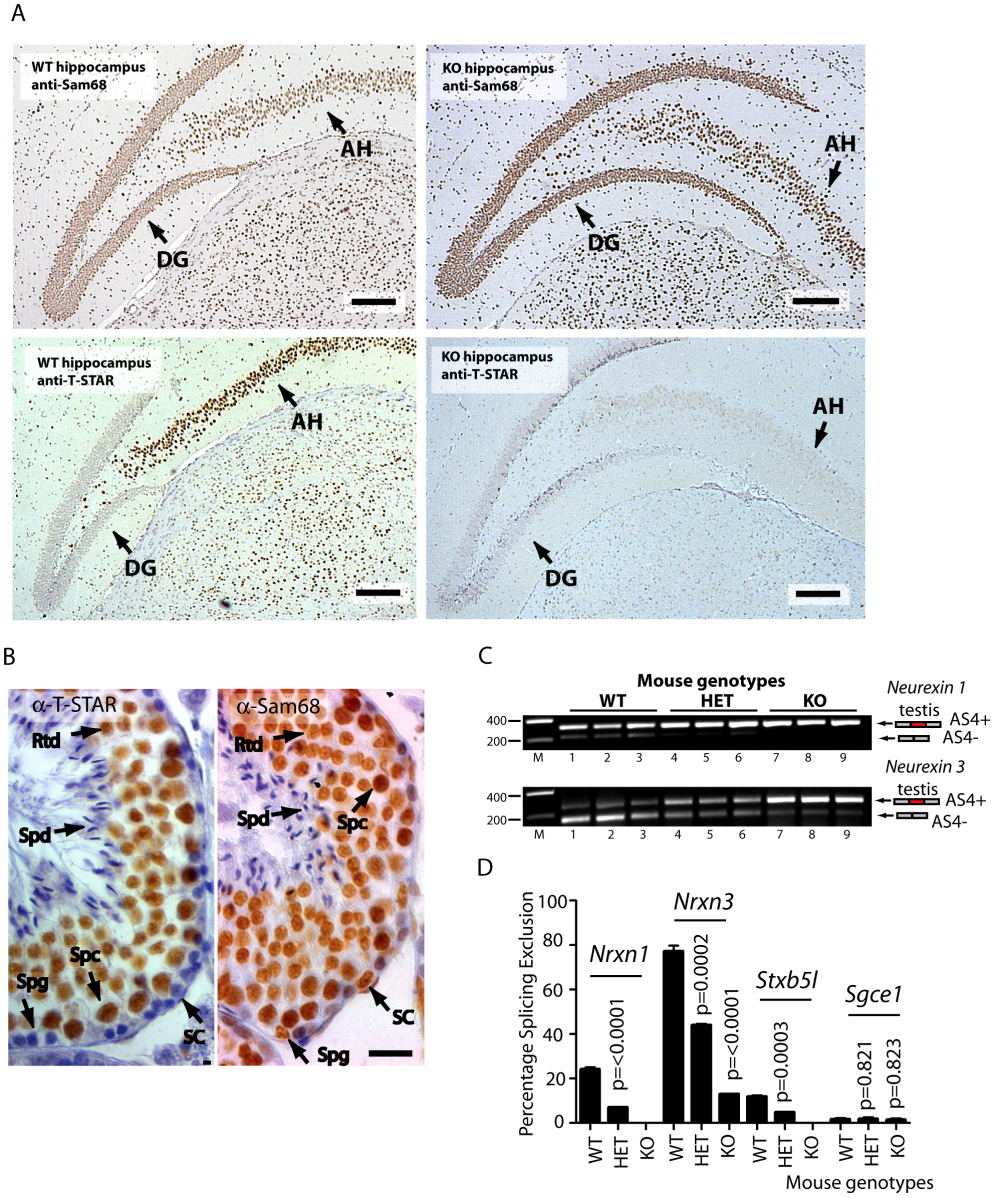
*Nrxn* exon AS4 alternative splicing control is dependent on the physiological expression of T-STAR protein even though Sam68 is co-expressed. (A) Immunolocalisation of T-STAR and Sam68 proteins in the mouse hippocampus from wild type or knockout mouse brains (Abbreviations: DG - Dentate Gyrus; and AH -Ammon's Horn). The scale bar is equivalent to 20 µm). (B) Immunolocalisation in the mouse testis. Paraffin embedded adult mouse testis sections were stained with affinity purified antibodies raised against T-STAR or Sam68 (brown staining), and counterstained with haematoxylin (blue). Abbreviations: Spg –spermatogonia; Spc –spermatocyte; Rtd –round spermatid; Spd –elongating spermatid; SC –Sertoli cell. The size bar corresponds to 20 µM. (C) Levels of *Nrxn1* and *Nrxn3* AS4 alternative splice isoforms in the testes of different mouse genotypes (n = 3 mice of each genotype) measured by RT-PCR and agarose gel electrophoresis. (D) Quantification of Percentage Splicing Exclusion in the testes of different mouse genotypes using capillary gel electrophoresis (n = 3 mice of each genotype: wild type mice *Khdrbs3^+/+^* (abbreviated WT) *Khdrbs3^+/−^* mice (abbreviated HET) and *Khdrbs3^−/−^* mice (abbreviated KO). The p values were calculated using unpaired t tests, to determine the significance of the difference between percentage splicing exclusion levels in the wild type versus either the heterozygous *Khdrbs^+/−^* mice (HET); or wild type versus the homozygous *Khdrbs3^−/−^* (KO) mice. The standard error of the mean is shown as an error bar.

We next addressed the question of whether T-STAR protein might function in the same or different cell types to Sam68. Strong nuclear expression of both T-STAR and Sam68 proteins were both detected in the CA1–CA3 regions of the hippocampus, with additional expression of Sam68 in the Dentate Gyrus (Figure 7A and Figure S3).

These experiments suggested overlapping patterns of expression of T-STAR and Sam68 in the hippocampus, but were unable to differentiate specific cell types. However immunohistochemical analysis of the testis clearly indicated that T-STAR and Sam68 proteins were co-expressed in exactly the same cell types and nuclei (in spermatocytes and round spermatids, with additional expression of Sam68 alone in spermatogonia and Sertoli cells Figure 7B). We could also detect *Nrxn1* and *Nrxn3* gene expression and AS4 exon skipping in the testis as well as in the brain (*Nrxn2* expression was only detected at very low levels in the testis, so we did not analyse it further here) (Figure 7C).

Although Sam68 was expressed in the same cell types as T-STAR in the testis, *Nrxn1* and *Nrxn3* AS4 splicing repression still critically depended on T-STAR protein expression (Figure 7C and 7D). *Nrxn1* AS4 splicing switched from a mean of 24% splicing exclusion in wild type testis to 0% splicing exclusion in the absence of T-STAR protein. In wild type testis the major *Nrxn3* mRNA isoform detected was the AS4-skipped form (with a mean value of 77% splicing exclusion). This splice isoform ratio was totally reversed in the *Khdrbs3^−/−^* background, where the major *Nrxn3* splice isoform now included AS4 (with a mean value of 13% AS4 splicing exclusion).

### *Nrxn2* AS4 is a specific molecular target for T-STAR regulation

*Nrxn1* AS4 is known to be a direct molecular target of Sam68 protein [27]. However, T-STAR-mediated regulation of *Nrxn2* was a surprise, since Sam68 protein had no reported effect at all on *Nrxn2* AS4 splicing regulation in mouse neurons [27]. We next tested if *Nrxn2* AS4 is indeed a direct and specific molecular target of T-STAR.

**Figure 8:**
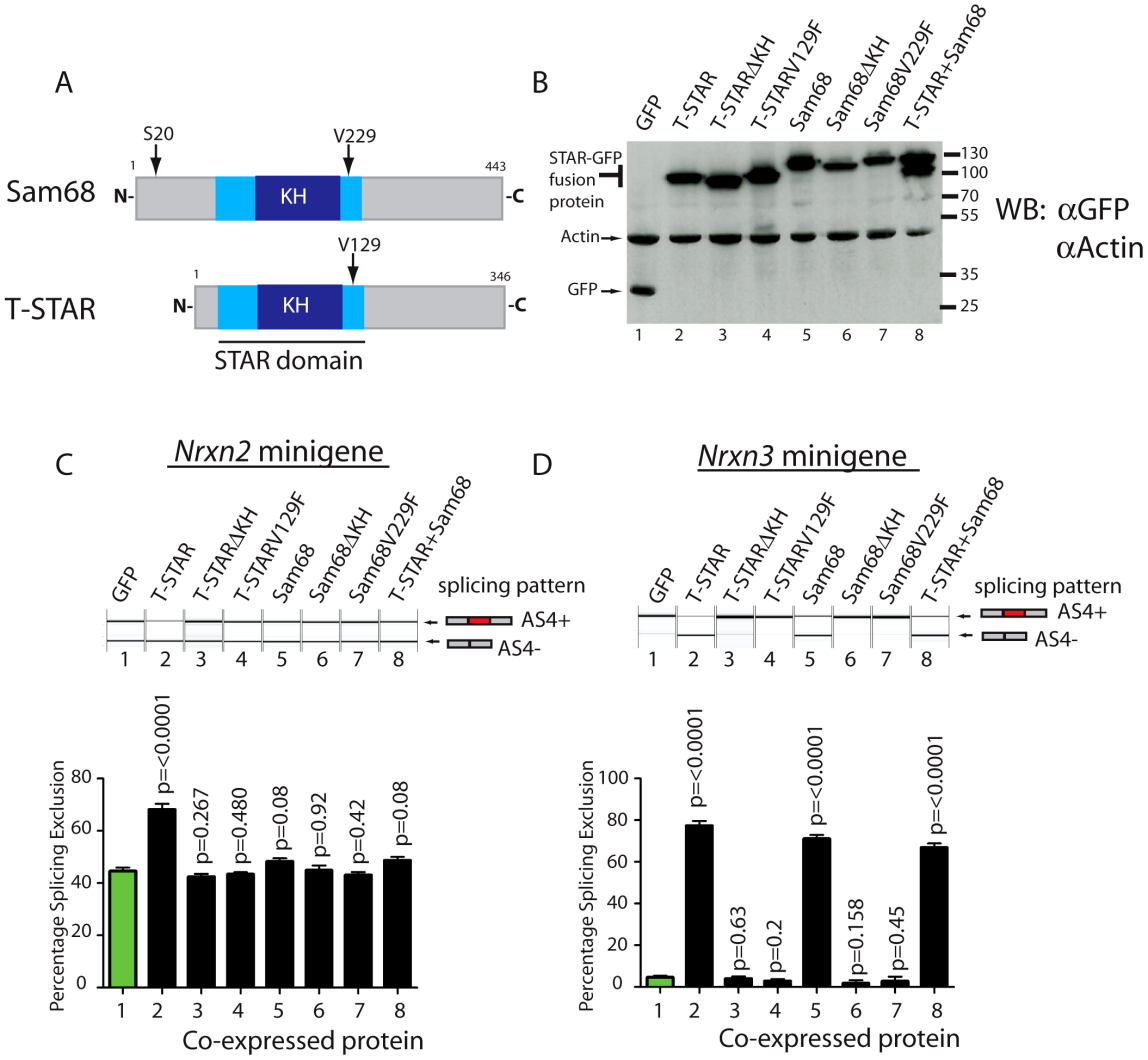
*Nrxn2* is a specific splicing target for T-STAR but not Sam68. (A) Comparative modular organisations of the Sam68 and T-STAR proteins. The position of the serine residue 20 (S20) in Sam68 which is phosphorylated to mediate splicing regulation of *Nrxn1* AS4 is indicated. (B) Representative Western blot analysis of HEK293 cells co-transfected with expression constructs for GFP-fusion proteins and minigenes. The Western blot was probed for GFP and actin, showing equal expression levels of each fusion protein. (C) *Nrxn2* AS4 is regulated by T-STAR but not Sam68. Representative capillary gel electrophoretogram (top) and bar chart (bottom) showing splicing data from single and three independent transfections respectively. (D) *Nrxn3* splicing is regulated by T-STAR and Sam68. Representative capillary gel electrophoretogram (top) and bar chart (bottom) showing splicing data from single and three independent transfections respectively. In each case, statistical significance was compared between HEK293 cells expressing GFP (lane1; shown as green bar) and HEK293 cells expressing the GFP fusion proteins (lanes 2–8 shown as black bars) using a non-paired t test and the error bar represents the standard error of the mean.

Minigene constructs were made containing mouse *Nrxn2* AS4 along with flanking intronic sequences. The resulting minigenes were co-transfected into HEK293 cells alongside constructs expressing either STAR-GFP fusion proteins or GFP alone. Parallel western blots showed that similar levels of the different proteins were expressed in transfected cells (Figure 8A–8B). Consistent with specific regulation by T-STAR, splicing exclusion of *Nrxn2* AS4 was only observed after co-transfection of an expression construct encoding T-STAR-GFP protein (Figure 8C, lane 2) and not by Sam68-GFP or GFP alone (compare lane 2 of Figure 8C with lanes 5 and 1).

We carried out similar minigene experiments to examine splicing regulation of the *Nrxn3* gene. Co-expression of either T-STAR-GFP or Sam68-GFP caused strong splicing repression of *Nrxn3* AS4 (leading to a mean of 70–80% splicing exclusion; Figure 8D, compare lanes 1, 2 and 5). Hence *Nrxn3* AS4 exon is regulated by both Sam68 and T-STAR. To examine whether or not the effect on splicing exclusion was due to direct RNA binding we tested the effect of the V229F amino acid substitution of Sam68 which has been reported to prevent RNA-protein interactions [43], and the corresponding V129F mutation in T-STAR (Figure 8A). Both these point mutations did disrupt splicing regulation by Sam68 and T-STAR (Figure 8C and 8D, lanes 4 and 7), as did deletion of the entire KH domain of either T-STAR or Sam68 (Figure 8C and 8D, lanes 3 and 6).

### An intronic A-U rich element downstream of the regulated *Nrxn2* exon mediates splicing changes response to cellular concentrations of T-STAR

**Figure 9:**
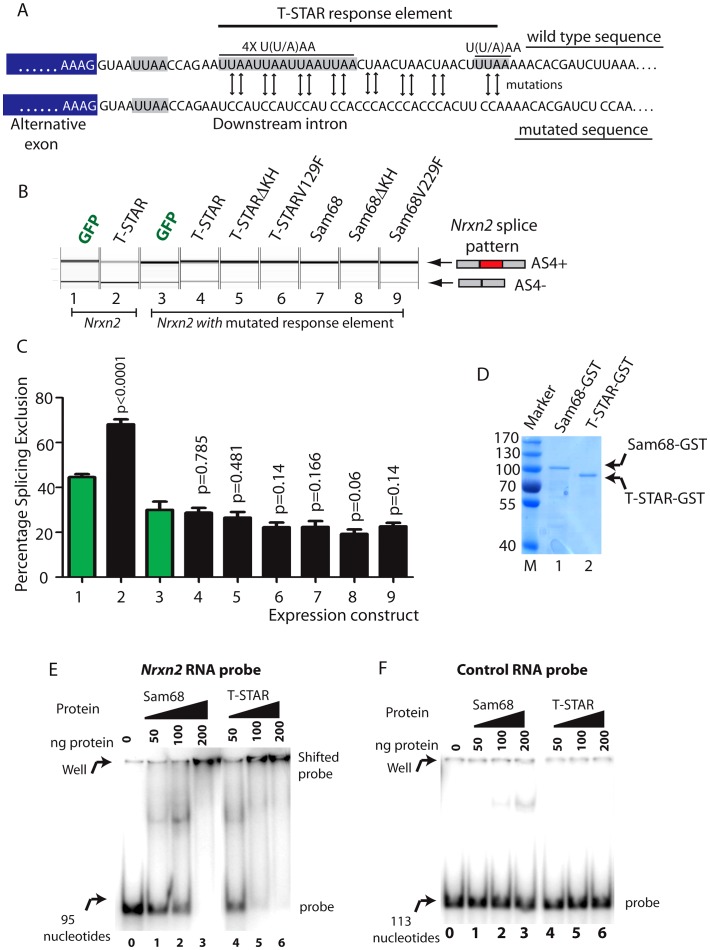
T-STAR mediates splicing repression of *Nrxn2* AS4 via a downstream response element. (A) Nucleotide sequence immediately downstream of the *Nrxn2* alternative exon (exon sequence shaded grey, intron sequence unshaded) of wild type gene and after mutagenesis to remove U((U/A)AA motifs. (B) Capillary gel electrophoretogram to show splicing response of mutated *Nrxn2* minigene after co-transfection with the indicated expression constructs. (C) Percentage Splicing Exclusion levels of the mutated *Nrxn2* minigene from 3 independent transfection experiments. The p values were calculated using unpaired t tests, and estimate statistical significance between splicing exclusion of the mutated *Nrxn2* minigene on co-expression of GFP(lane 1: shown as green bar) and each of the individual fusion proteins (lanes 2,4–7: shown as black bars). (D) Coomassie stained gel showing purified Sam68-GST and T-STAR-GST fusion proteins used for gelshift experiments. (E) EMSA experiment using RNA probe containing the *Nrxn2* response element. (F) EMSA experiment using a control RNA probe.

We next set out to identify the RNA sequences which mediate splicing repression of T-STAR protein on *Nrxn2* AS4. SELEX experiments have identified bipartite U(U/A)AA (abbreviated UWAA) motifs as T-STAR RNA target sites [44]. In the overall length of the *Nrxn1* and *Nrxn3* genes UWAA motifs occur at a lower frequency than would be expected by chance, but there was an excess in the *Nrxn2* gene, particularly of the double repeat UWAAUWAA in the region of the *Nrxn2* AS4 exon (Table S1). Detailed analysis of the AU-rich region downstream of *Nrxn2* AS4 revealed six candidate UWAA T-STAR target motifs within a 51 nucleotide AU-rich region which starts 13 nucleotides downstream of *Nrxn2* exon AS4 (Figure 9A) [9].

We altered five of the UWAA motifs downstream of *Nrxn2* AS4 using mutagenesis (the most upstream of the UWAA sequences was close to the 5′ splice site so we did not alter this), and then examined the effect on splicing regulation by T-STAR. Importantly, the alternative exon in the *Nrxn2* minigene was still efficiently spliced into mRNAs after mutagenesis, indicating the *Nrxn2* exon itself was still efficiently recognised by the spliceosome (Figure 9B–9C, Lane 1). Hence no essential splicing signals had been compromised by the mutations engineered into the minigene construct. However, mutation of the UWAA repeats completely prevented splicing repression by T-STAR of *Nrxn2* AS4 (Figure 9B–9C). We also observed reduced levels of splicing exclusion for the mutated *Nrxn2* AS4 minigene compared to the wild type after co-transfection with GFP (significantly different mean levels of percentage splicing exclusion were observed between lanes 1 and 3, p = 0.0079), consistent with this UWAA repeat acting as an intronic splicing silencer responsive to endogenous T-STAR protein in the HEK293 cells.

We carried out EMSAs (Electrophoretic Mobility Shift Assays) using equal concentrations of purified T-STAR-GST and Sam68-GST proteins (Figure 9D) to confirm direct RNA-protein interactions with the *Nrxn2* response element. Addition of either T-STAR or Sam68 completely prevented the *Nrxn2* RNA probe from moving from the well, suggesting the formation of large molecular complexes on this probe. T-STAR protein bound to and shifted the *Nrxn2* probe (Figure 9E, lanes 4–6) with a complete shift observed with 100 ng of added T-STAR protein (lane 0 in Figure 9E shows how the probe migrates in the absence of T-STAR or Sam68 protein). Sam68 protein also shifted the *Nrxn2* RNA probe, but with a different response pattern of maximal binding at 200 ng added protein (Figure 9E, lanes 1–3). These different patterns of binding response might contribute to the specific regulation of *Nrxn2* AS4 by T-STAR protein *in vivo*. No binding was observed to a control RNA probe (Figure 9F).

**Figure 10:**
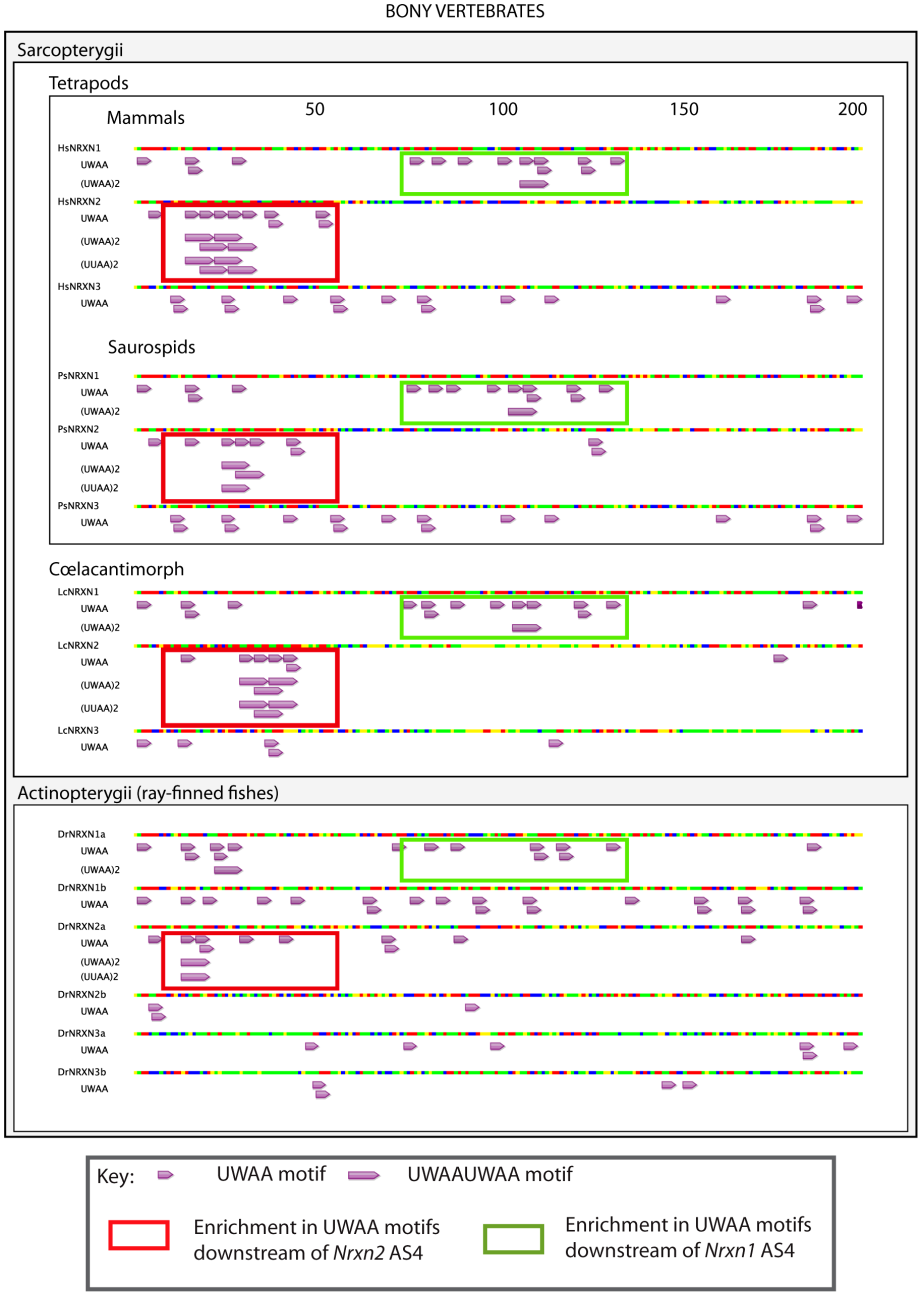
Distinct arrangements of UWAA motifs are shared between *Neurexin* gene paralogs in bony vertebrates and predict an ancient mechanism of splicing control. Annotation of UWAA motifs in the 200 nucleotide regions downstream of AS4 in human (abbreviated Hs); tortoise (abbreviated Ps); Ceolocanth (abbreviated Lc); and zebrafish (abbreviated Dr). The *Nrxn* genes have duplicated in the zebrafish, and these two copies are shown distinguished as the *Nrxn1-3*a and *Nrxn1-3*b copies. Nucleotides are shown as red (A), green (U), blue (C) and yellow (G). Concentrations of UWAA motifs are boxed as indicated in the key.

The presence and organisation of UWAA motifs in the 200 bp downstream of the AS4 exon in *Neurexin2* genes were highly conserved between bony vertebrates (Figure 10), suggesting that the T-STAR response element is ancient. Although different from *Nrxn2*, the positions of UWAA motifs downstream of the *Neurexin1* AS4 exon were also highly conserved between individual *Nrxn1* genes in bony vertebrates. There was less conservation of UWAA distribution downstream of the *Neurexin3* AS4 exon.

**Figure 11:**
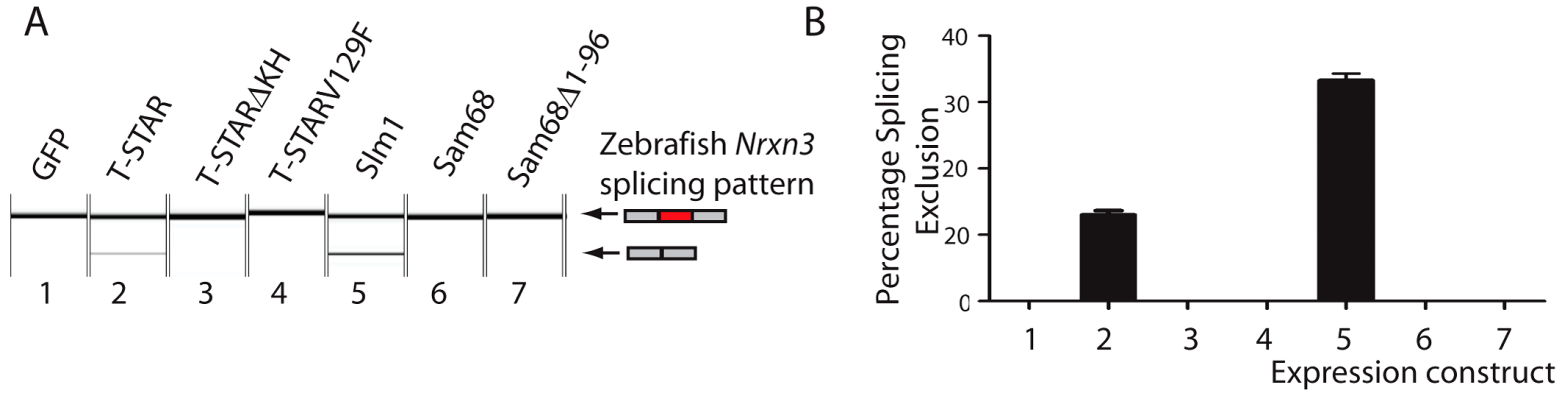
Human T-STAR protein represses splicing inclusion of the Zebrafish *Nrxn3* AS4 exon. (A) Capillary gel electrophoretogram showing splicing of a minigene encoded zebrafish *Nrxn3* in response to co-expressed proteins introduced by co-transfection. (B) Quantification of biological replicates from three independent co-transfection experiments.

Alternative splicing of *Nrxn1-3* AS4 has previously been found in zebrafish showing it is ancient in origin [45]. Since the T-STAR gene itself originated 520–610 million years ago (Figure S1) and the *Neurexin* genes diverged about the same time (data not shown) we carried out experiments to test if T-STAR might also control *Neurexin* AS4 splicing in zebrafish. We tested this hypothesis using the zebrafish *Nrxn3* (abbreviated z*Nrxn3*) AS4 exon, since the mouse *Nrxn3* AS4 exon was under the tightest regional control in the mouse brain, and also very strongly repressed by the presence of either T-STAR or Sam68 proteins when encoded by a minigene. Consistent with a conserved mechanism of splicing regulation, a minigene-encoded z*Nrxn3* AS4 exon was strongly repressed by co-transfection of human T-STAR protein (Figure 11A and 11B, lane 2). No splicing repression was induced by either the V129F T-STAR mutant, or by the T-STAR ΔKH domain mutant. Splicing inclusion of the z*Nrxn3* AS4 was also repressed by human SLM-1 protein, but not by Sam68 (Figure 11A and 11B, lanes 5 and 6). Zebrafish Sam68 lacks the N-terminal extension of human Sam68 protein [16], but even deletion of these 96 amino acids from human Sam68 protein did not enable human Sam68 protein to regulate z*Nrxn3* AS4. Figure 11A and 11B, lane 7)

### T-STAR null mice have normal spatial memory in the absence of AS4 exon repression in the hippocampus

**Figure 12:**
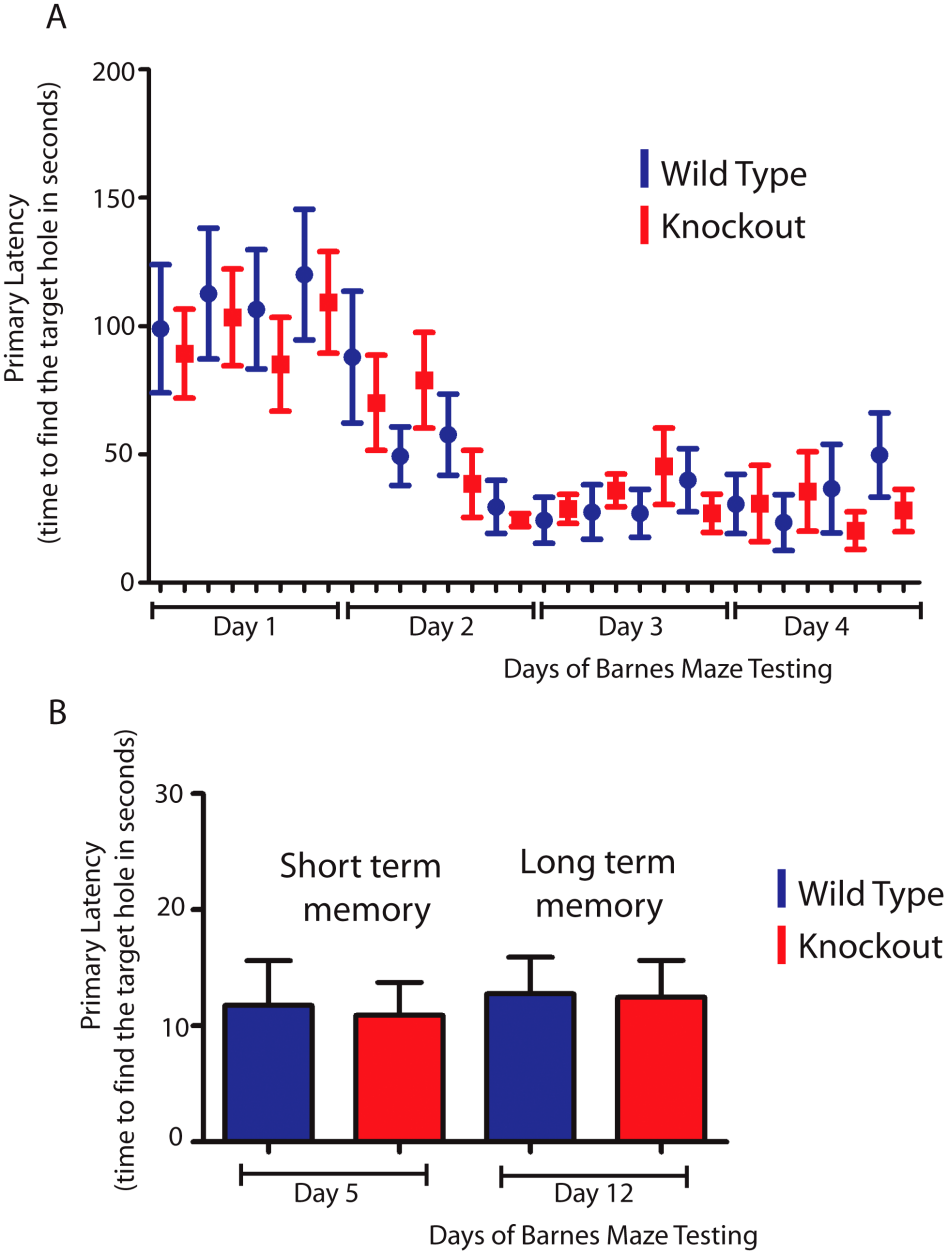
T-STAR null mice have long and short term spatial memory equivalent to wild-type mice. (A) Wild type and knockout mice were trained each day(four trials) for four days in a Barnes maze, and the time taken to find the escape hole (primary latency) was monitored. (B) Time taken to find the escape hole on day 5 (short term memory) and on day 12 (long-term memory)with no training between day 5 and day 12 (n = 8 wild type and 11 knockouts and error bars indicate the standard error of the mean).

Since the hippocampus is involved in spatial learning and memory [46] we hypothesized that T-STAR null mice with reduced levels of the AS4 exon negative *Neurexin* isoforms might show differences in either spatial learning or memory. To test this we used a Barnes maze test to measure how well T-STAR null mice remember the spatial location of an escape route (hole) using visual cues. The T-STAR knockout mice and the wild type mice both learned the spatial acquisition task equally well over a period of four days (Figure 12A) and had similar short and long term memories measured at 5 and 12 days respectively (Figure 12B). Therefore in these mice no difference in learning was observed, despite significantly less splicing repression of *Neurexin* AS4 exons in the hippocampus.

## Discussion

**Figure 13:**
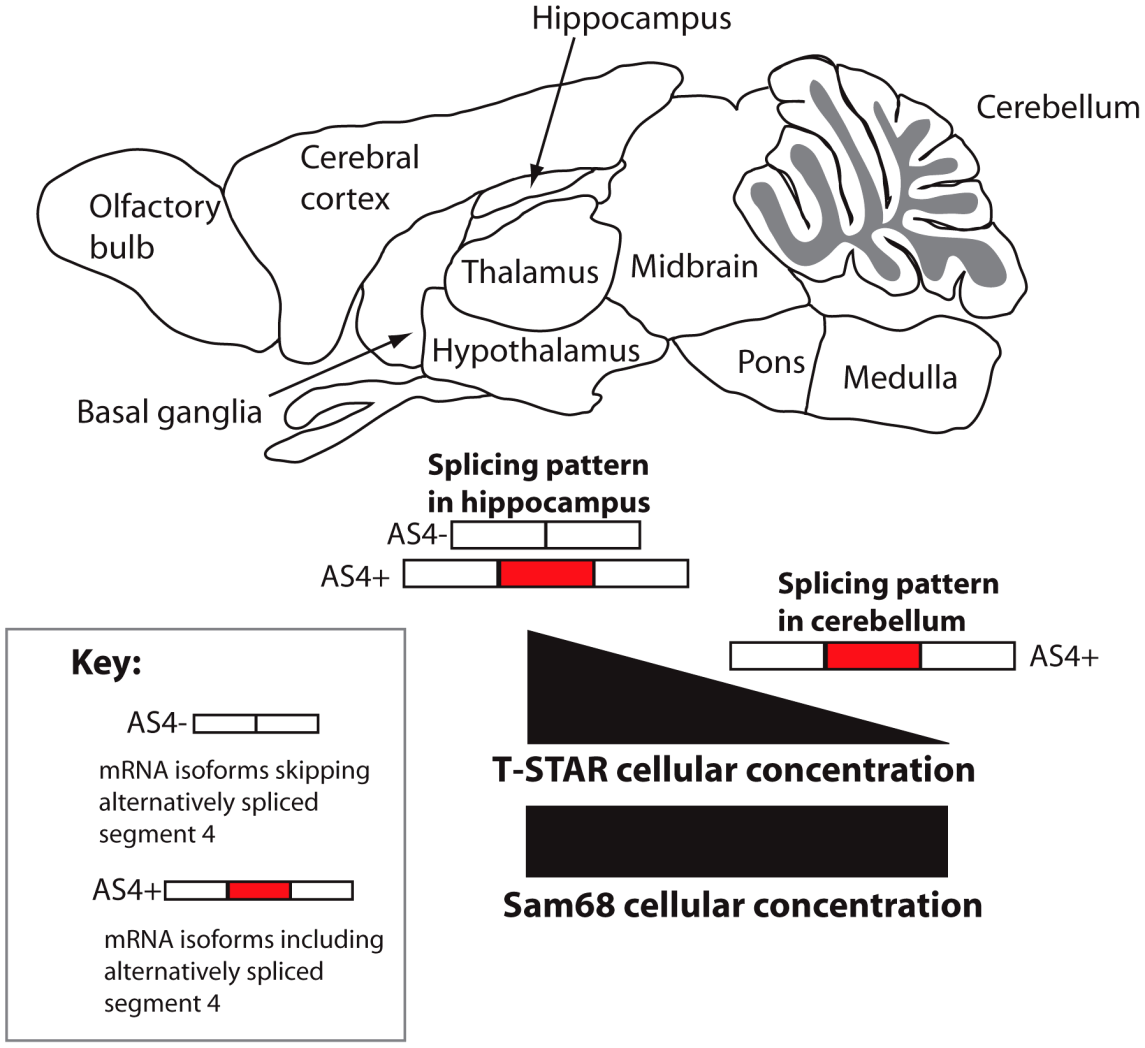
Concentration-dependent splicing model for regional regulation of *Nrxn1-3* AS4 in the mouse brain. T-STAR protein directly regulates *Nrxn1-3* AS4 splicing. In the cerebellum T-STAR concentrations are low and most of the *Nrxn1-3* mRNA isoforms include AS4 as a result. In the forebrain-derived regions T-STAR concentrations are high, and there are also increased levels of AS4 splicing exclusion resulting from this. Sam68 protein levels are similar across the brain regions.

A distinct T-STAR gene has been maintained in bony vertebrates for at least 550 million years, ever since the gene triplication which also produced the genes encoding Sam68 and SLM-1. Here we have identified for the first time (to the best of our knowledge) the molecular function of endogenous T-STAR protein, which is to control regional splicing repression of the AS4 exon in the *Nrxn1-3* mRNAs. T-STAR also controls splicing regulation of the *Syntaxin-binding protein 5-like* (*Stxbp5l*, alternatively known as *Tomosyn2*). Together our data support a model in which T-STAR expression provides a concentration-dependent switch to establish *Nrxn1-3* AS4 splicing patterns in different regions of the mouse brain (Figure 13). High concentrations of T-STAR in forebrain-derived structures like the hippocampus block splicing inclusion of *Nrxn1-3* AS4. Lower concentrations of T-STAR protein in areas of the brain like the cerebellum result in the *Nrxn1-3* AS4 exons being mainly included. Three lines of evidence support this mechanism. Endogenous *Neurexin* AS4 splicing patterns responded to T-STAR protein concentration differences found between wild type, heterozygous and homozygous knockout mouse brains. Second, local endogenous levels of T-STAR protein expression in the brain showed good correlation with the regional patterns of *Neurexin* AS4 splice isoforms. Thirdly, removal of T-STAR protein in the null mouse totally blocked regional *Neurexin* AS4 alternative splicing patterns, even though Sam68 was still there.

The T-STAR parolog Sam68 also regulates alternative splicing of the *Nrxn1* AS4 exon, but in this case splicing repression involves neuronal signalling pathways [27]. T-STAR protein lacks the key serine residue (S20) which is phosphorylated by these neuronal signalling pathways (Figure 8A). Deletion of Sam68 predominantly affected regional *Nrxn1* AS4 splicing repression in the cerebellum and brain stem, with very slight effects on *Nrxn1* AS4 splicing repression in the cortex [27]. In contrast, T-STAR has strong splicing effects on AS4 inclusion in all forebrain-derived regions of the adult brain which are also the sites of maximum AS4 splicing repression, and where Sam68 does not seem to be so active. Our data also show that T-STAR controls *Nrxn1* and *Nrxn3* AS4 splicing in the testis, which does not contain neuronal tissues and directly co-expresses Sam68 in exactly the same cells as T-STAR. Despite this splicing defect, T-STAR null mice did not have any major defects in germ cell development. This is again in direct contrast with Sam68 null mice, which suffer germ cell arrest and infertility.

We have also identified *Nrxn2* exon AS4 as the first known splicing target for T-STAR protein which is not also regulated by Sam68. The T-STAR response element in *Nrxn2* AS4 mapped to six repeated UWAA motifs which would be predicted to bind to T-STAR protein by SELEX [44]. T-STAR protein operates as a splicing repressor of *Nrxn2* AS4. Although downstream binding sites for splicing regulators frequently cause exon activation rather than repression, the AU-rich sequence responsible for T-STAR mediated splicing repression is very close (12 nucleotides) to the 5′ splice site of *Nrxn2* AS4. Binding of T-STAR protein to this region of the pre-mRNA might mechanistically repress splicing through exclusion of U1 snRNP [47]–[49]. The presence of multiple UWAA binding sites downstream of *Nrxn2* AS4 may ensure that at least a single site is occupied at a given cellular concentration of T-STAR, or help assemble larger protein-RNA complexes [50].

Each of the Neurexin proteins is somewhat similar, which may provide a physiological rationale for their coordinate regulation by a single master protein like T-STAR. However, different distributions of UWAA motifs downstream of individual AS4 exons in different *Neurexin* gene paralogs suggest subtly different splicing control mechanisms operate. These patterns of UWAA motifs were conserved between *Neurexin* gene paralogs in different species. Different patterns of *Neurexin* AS4 splicing exclusion were also observed between *Neurexin* paralogs in the mouse brain, with *Nrxn3* AS4 having a much tighter pattern of regulation than the equivalent exon in *Nrxn1* or *Nrxn2*.

The AS4 exons of the *Neurexin* genes are ancient, and conserved even in zebrafish [45], indicating an important function for this splice isoform. Moreover, in transfected cells human T-STAR protein was also able to repress splicing of zebrafish *Nrxn3* AS4, suggesting splicing control by T-STAR is both ancient and conserved and may have been one of the earliest functions for T-STAR protein after it evolved. Neurexin proteins play important roles in synapse function and guiding wiring of the nervous system, and have been implicated with roles in Alzheimer's disease, autism and epilepsy [51]. The AS4 exon has been suggested to play a critical role in moulding the synapse [39]–[41]. Nonetheless, even though T-STAR null mice almost totally fail to repress splicing of the *Neurexin* AS4 exon in the embryo as well as the adult they still develop apparently normal brains and have normal spatial memory measured by the Barnes maze test. Taken as a whole these results suggest the functional effect of the AS4 exon might be somewhat subtle, yet must be important in the wild to explain the conservation of this alternative splice event in bony vertebrates.

Perhaps the most surprising implication of the results described in this study is the exquisite and unexpected specificity of the effects of T-STAR on alternative splicing regulation. While we sampled 782 alternative splicing events known to be differentially regulated in the mouse brain, we only identified 4 strongly regulated splicing targets. At the very least our data suggest an enrichment of T-STAR targets involved in synapse formation, and is consistent with the idea that T-STAR, like some other RNA binding proteins, will functionally regulate coherent groups of targets [52]. Very recent data indicate that the neurexin and tomosyn proteins are involved in the mechanism of synaptic retrograde transport inhibition in *C. elegans* [53], consistent with functional coherence in their shared splicing regulation by T-STAR.

## Materials and Methods

### Ortholog identification

Blastp and tblastn searches for neurexin and KHDBRS orthologous sequences in nr, reference genomic sequences, reference mRNA and EST databases were performed using the NCBI Blast suite (http://blast.ncbi.nlm.nih.gov/). Accession numbers for NRXN and STAR protein sequences used are listed in Dataset S2. Human, chick and fish genes are respectively ENSG00000179915, ENSGALG00000009107, ENSDARG00000061647 (NRXN1); ENSG00000110076, ENSDARG00000061454 (NRXN2); ENSG00000021645, ENSGALG00000010518, ENSDARG00000062693 (NRXN3).

### Phylogenetic inference

Trees were inferred by using MrBayes [54] and PhyML [55]. Neurexin or KHDBRS sequences were aligned with MAFFT [56]. Due to sequence variability in the COOH ends, we only used the GSG domain [57] for further analysis. For clarity, we restricted the sampling to specific taxons (Mammals [human, rat], Birds [chick], Amphibians [frog], Bony fishes [zebrafish], Jawless fishes [lamprey], Urochordates [sea squirt, appendicularians], Cephalochordates [lancelet], Echinoderms [sea urchin], Hemichordates [acorn worm], Mollusks [sea hare], Ecdyzozoans [nematode, drosophila, mosquitos, honey bee], Cnidarians [hydra, sea anemone], Placozoans [trichoplax], Choanoflagellates [monosiga]). Alignments were analyzed with ProtTest (v. 10.2) to identify the best substitution models [58]. We used MrBayes 3.1.2 with the wag matrix rate and a gamma distribution describing among-site rate variation with eight categories (+G8). MCMCMC chains were run for 1 million generations with a sample frequency of 1,000 and a 10% burn-in value. For ML analyses, we also used the wag+G8 in PhyMLM while searching for the ML tree by performing both NNI and SPR topological moves on a bioNJ starting tree. The statistical robustness of inferred nodes was assessed by 100 bootstrap pseudoreplicates of the same ML search. Whatever the method, trees inferred showed same node support. We used the SF1 family as an external outgroup, since it is the only GSG protein family found in unicellular eukaryotes (e.g. *M. brevicollis*). Analyses were conducted using the Geneious Pro package (v5.6, available from http://www.geneious.com) [59]. The significance of deviations in UWAA motifs within *Neurexin* genes were measured using the R'MES program as described [60].

### Detection of gene and protein expression in mouse tissues

PCR reactions (Dataset S1) were designed to detect alternative mRNA isoforms in the mouse transcriptome, including all the simple alternative splicing events in the mouse RefSeq database NCBI genome build 37 (UCSC mm9) using gene annotation from UCSC known gene track as of 2009/09/01. Initial medium throughput analysis was carried out on a single whole brain RNA sample from wild type and knockout mouse brain, using a robotic platform as previously described [42] to assay 1191 ASEs between wild type and knockout whole brain mRNA with size differences between the two expected isoforms between 30 and 411 bases. The ASEs included 808 alternative events exons, 129 alternative 3′ splice sites, 155 alternative 5′ splice sites and 99 more complex alternative splicing events. Subsequent quality control removed 141 assays that gave no PCR products, 142 assays that gave impure PCR reactions with less than 75% of products at the required mobilities, and 115 assays that gave weak products which had less than 20 nM total concentration. Out of the 792 events that gave informative splicing ratios only 20 alternative splicing events changed more than 16% between wild type and knockout adult mouse brains, and just 7 exons showed a greater than 25% difference in splicing inclusion between the wild type and knockout mouse brain. Of these 7 exons we confirmed just 4 (in the *Nrxn1-3* genes and *Stxbp5l*) in the brains of multiple replicate mice.

The levels of *Nrxn1-3* AS4 isoforms were detected in total RNA isolated from different mouse tissues using RT-PCR and standard conditions [61] using previously described primers [27]. Quantifications were carried out by Capillary Gel electrophoresis as previously described [61], [62].

Northern analysis was carried out using standard techniques. T-STAR mRNA was detected using a PCR probe amplified from the T-STAR cDNA using the primers TstarN F 5′GCCACTTTGTTGAAGCATCC3′ and T-STARNR 5′ AAATTCTATGGAAACCTTTAAG 3′, and the was blot re-probed using 18S RNA as a loading control [63].

For protein detection by immunohistochemistry, testes and brains were fixed in 4% paraformaldhyde and embedded in paraffin wax. Sections were prepared and immunohistochemistry carried out as previously described [64]. Primary antibodies were specific for Sam68 (Santa Cruz anti-Sam68 sc-333) and affinity purified α-T-STAR [22], [65]. Protein detection by Western blotting was as previously described [22], using antisera specific to either Sam68 (Santa Cruz sc-333) or T-STAR [22], [65] protein. To detect protein levels across the mouse brain, blots were first probed for Sam68, and then these same blots sequentially stripped and re-probed for T-STAR. The western blot shown in Figure 1 was probed with the α-Khdrbs3 antibody (Proteintech 13563-1-AP) which recognises both T-STAR and Sam68.

### Statistical analysis

Bar charts were plotted and statistical analyses performed using Graphpad Prism (Graphpad software).

### Construction of targeting vector for the *Khdrbs3* gene

We constructed a targeting construct ELD1-HR in which exon 2 of the mouse *Khdrbs3* gene was flanked by *Lox*P sites using standard molecular biology techniques. Three overlapping fragments from the *Khdrbs3* locus were initially amplified by long range PCR from 129Sv/Pas isogenic DNA. The primers 5′-GCCTCAAAGGTGGTTATGTCCTCTGG-3′ and 5′-AAATCACTGAGCCCTTGGGTGACC-3′ were used to create ELD1-Lad (long arm distal fragment). The primers 5′ -TTGTCTCGCTCTCTAGGTTCTCTCCTGG-3′ and 5′- GGTTTCTCAAGCATCCACAAGCATACG -3′ were used to create ELD1-Lap (long arm proximal fragment). The primers 5′-AGCTGGGACAGAAGGTGCTGATTCC-3′ and 5′- TGCACCACAATAAGATAGCCCAGCC-3′ were used to create ELD1-Sam (short arm fragment). These products were then independently cloned into the pCR4-TOPO vector (Invitrogen) and sequenced. ELD1-Lad contains intronic sequence 5′ of exon 2. ELD1-Lap contains sequences both upstream and downstream of exon 2 and also includes exon 2. ELD1-Sam has part of exon 2 and some intronic sequence between exon 2 and 3.

To make a positive control for the ES cell electroporation (construct ELD1C+) the G139 vector containing one *Lox*P site and neomycin flanked by *Frt* sites was modified so that the *Bsa*BI-*Bsu*361 fragment from ELD1-Sam could be cloned into it. An adapted cloning vector was made to clone the long arm of the targeting construct. A linker was synthesized containing *Asc*I, *Not*I, *Sac*II, *Bsm*I, *Hind*III, *Afe*I, *Mlu*I, *Pci*I, *Avr*II, *Xho*I, *BstE*ii, *Nru*I and *Pac*I sites, and inserted into the G126 vector to create the construct ELD1-GA1. A *Bsm*I –*Hind*III fragment from ELDL1-Lad was then cloned into the *Bsm*I-*Hind*III site of ELD1-GA1. The *Hind*III-*Bsa*B1 from ELDL1-Lap was then inserted to create the construct ELD1-LA. A *Lox*P site was cloned into the *Hind*III site using two annealed oligonucleotides to create the construct ELD1-LA-Lox.

An *XhoI*-*Bst*II fragment from ELD1C+ was cloned into ELD1-GA1 to create the clone ELD1-SA Neo. Next the *Sac*II-*Mlu*I fragment of ELD1-LA was cloned into ELD1SAneo. This construct (ELD1-LSAneo) contained the long and short arms. The last step was to insert the Diptheria toxin selection cassette from the G112 vector into the *Asc*I-*Not*I site of ELD1-LSA to create the final ELD1-HR targeting vector.

### Generation of knockout mice

The ELD1-HR targeting vector was electroporated into 129Sv/Pas cells by Genoway,France, and clones were screened using the primers GX1406 5′-CTACTTCCATTTGTCACGTCCTGCACG-3′ and ELD1J2 5′-ACAGCCACCCCACACTCAGAAACG-3′. We obtained a targeting frequency of 34%. Positive clones were injected into blastocysts (by Genoway,France) to create chimeras and bred to yield agouti pups heterozygous for the targeted locus by PCR and Southern blot analysis. After germline transmission of the conditional allele was achieved, we confirmed the genotype of these mice by Southern blot. The original mice containing the *Neomycin* gene were crossed to FlpE mice to remove the Neo gene resulting in the *Khdrbs3^LoxP^* allele depicted in Figure 1C. Mice containing the *Khdrbs3^LoxP^* allele were crossed to mice expressing PGK-cre, resulting in the deletion of *Khdrbs3* exon 2 (Figure 1D). Genetic structures of the targeted and wild type alleles were confirmed by Southern blot analysis using the SA-E-V probe generated by PCR amplification with the primers SA-E-V1F 5′- TGTCAACCAGAGGACAGTAGAGGACTCACC-3′ SA-E-V2R 5′- GCCCTCATGTTGGAAGGAACCACC-3′ (Figure 1B–D), and *Sac*I/*Avr*II digested mouse genomic DNA. Levels of *Khdrbs3* gene expression were monitored at the RNA level using RT-PCR using primers:

Tstar exon1F 5′-GCGAGCATGGAGGAGAAGTA-3′;

Tstar exon3R 5′- CTTTGCCAAGGATGGACATT-3′;

HrptF 5′-CCTGCTGGATTACATTAAAGCACTG-3′; and

HprtR 5′-GTCAAGGGCATATCCAACAACAAAC-3′

### Analysis of mouse germ cell development *in vivo*

Litter sizes, testis/body weight ratios, sperm counts and Mendelian ratios were measured on a mixed C57Bl6/129 background and a Bl/6 background. In order to determine sperm counts, the cauda epididymis were dissected in Universal IVF media (Origio, Surrey) and the sperm were counted in a haemocytometer.

### Analysis of exon splicing using minigenes

The *Nrxn2* and *Nrxn3* minigenes were constructed by PCR amplification of mouse genomic DNA, followed by cloning into the exon trap vector pXJ41 as previously described [61], [66]. The primers used for PCR amplification were:

Mouse *Neurexin*2F 5′-AAAAAAAACAATTGgtgaggagatggctgggact-3′;

Mouse *Neurexin*2R 5′-AAAAAAAACAATTGaaaaacccctgaggtgaactct-3′;

Mouse *Neurexin* 3F 5′-AAAAAAAACAATTGaaaaaggacgaggaggagttt-3′;

Mouse *Neurexin* 3R 5′-AAAAAAAACAATTGtcttagactttttgagttgacttgatg-3′.

Zebrafish *Neurexin* 3F 5′-AAAAAAAACAATTGtggagaaaaactgaagaaaatgaa-3′.

Zebrafish *Neurexin* 3R 5′-AAAAAAAACAATTGctaactttaagatcaacacaaagatca-3′.

The T-STAR response element in *Nrxn2* was mutated by overlap PCR, using the following mutagenic oligonucleotide primers:

Mutant*Nrxn2*F 5′-AatCCatCCatCCatCCacCCacCCacCCacttCCaaaacacgatctCCaaggtgcagagctctctc-3′.

Mutant*Nrxn2*R 5′-GGagatcgtgttttGGaagtGGgtGGgtGGgtGGatGGatGGatGGatTctggttaattacctttgtc-3′.

Levels of alternative splicing were detected using RT-PCR and capillary gel electrophoresis as previously described [61], [66].

The V129F mutant of T-STAR, V229F mutant of Sam68, (KH version of T-STAR, and (KH version of Sam68 were cloned by overlap PCR mutagenesis as previously described [67] using the following primers:

T-STARmutagenesisF: 5′-gttctcattgaaTtTtttgccccacctgcagaagctt-3′

T-STARmutagenesisR: 5′-aagcttctgcaggtggggcaaaAaAttcaatgagaac-3′.

T-STAR(KHF: 5′-ttcaactttgtggggaaa gagttgaggaaaagtggagaa-3′

T-STAR(KHR: 5′-ttctccacttttcctcaactctttccccacaaagttgaa-3′

Sam68 mutagenesisR: 5′-tcaatgaagaaatgcagatcc-3′

Sam68 mutagenesis F: 5′-ggatctgcatttcttcattga-3′

Sam68ΔKHF: 5′-tgtcaagcagtatcccaaggagctgcgcaaaggtgg-3′

Sam68ΔKHR: 5′-ccacctttgcgcagctccttgggatactgcttgaca-3′

Final PCR products after overlap PCR were each cloned into pGFP3 to generate the expression constructs [22].

### Electrophoretic mobility shift assays (EMSAs)

EMSAs were performed as previously described [61], [62] using purified full length Sam68-GST and T-STAR-GST fusion proteins, and in vitro transcribed RNA probes made from regions of the *Neurexin2* gene cloned into pBluescript. The sequences of the inserts of the pBluescript clones were:

### *Nrxn2*

GGCTGCTCGACAAAGGTAATTAACCAGAATTAATTAATTAATTAACTAAC

TAACTAACTTTAAAAACACGATCTTAAAGGTGCAGAGCTCTCTCC

### Control probe

AGGCCCCCTAGAAGTAGTGCAGGCTGGTGGCTGACCGGACCAAGGGAGGA

AGGGAAGGTGGTGCTCTCTTAGGAATCCATAGAGGTCTCTGCCTGCTGGT

TTGATGAGGAAGA

### Barnes maze test

The Barnes maze behavioural experiment was performed as described (http://www.nature.com/protocolexchange/protocols/349). Eight Bl/6 wild type and eleven T-STAR knockout male mice (10 weeks old) which had been backcrossed >10 generations onto a Bl/6 background were used for the analysis. The maze consisted of 20 holes, with one target hole which has a box into which the mouse can escape from the light shone on the maze. Mice were trained using visual cues to find the target hole over a period of 4 days with 4 trials each day. We measured the primary latency (or time to find the hole in seconds). On the fifth day, the box was removed and the time to find the target measured to determine short term memory. The mice were allowed to rest for 7 days and then they were tested again to monitor their long term memory on the twelfth day. All animal experiments were performed with approval from Newcastle University ethical review committee and under UK home office licence according to the requirements of the Animals (Scientific Procedures) Act 1986 of the UK Government.

## Supporting Information

Dataset S1 Complete dataset from transcriptome-wide analysis of splicing in the wild type and *Khdrbs3^−/−^* brain, including details of the 792 alternative splicing events in our screen. Columns: a. Gene name; b. splicing event type; c–e splicing event details; These columns give 150 nucleotides of upstream and downstream intronic sequence plus the exon sequence for cassette exons. For alternative 5′ and 3′ sites the two splice sites and adjacent sequence are given; f–i gives the primer sequences and short and long product sizes expected; j and k give the percentage spliced in (psi) values for the wild type and KO brain. Psi = concentration of the long form dividend by the sum of the concentrations of the long and short forms (values given as percentages). l gives the difference in percent spliced in values between wild type and ko brain. The targets are ordered according to this shift; note the *Neurexin* genes in position 1 and 2.
(XLSX)

Dataset S2 Accession numbers for sequences used to build the cladograms in Figure S1 and and to analyse the comparative genomics of the *Neurexin* genes in Figure 10.
(XLSX)

Figure S1 The *Khdrbs3* gene evolved via gene triplication early in the radiation of bony vertebrates. Cladogram indicating the evolutionary profiles of the KHDRBS proteins, using the STAR protein SF1 as an external outgroup since SF1/MSL5p is the only GSG protein found in unicellular eukaryotes like *S. cerevisiae*. The KHDBRS proteins form a well supported monophyletic group, in which members are already present in early metazoans (Hydra, Trichoplax). Drosophila KEP1 and related proteins belong to this group. Whereas cephalochordates and insects code for KHSRBS-related sequences, no homolog was found in *Ciona intestinalis* nor in *C. elegans*, indicating a secondary loss of Sam68 genes in these taxons. Blast analysis of the lamprey genome suggests that it encodes only 4 STAR proteins. One is SF1, the other three derive from lamprey-specific duplications of a single KHDBRS gene. The three subgroups T-STAR, Sam68 and SLM-1 (*KHDBRS1*, *2* and *3* genes) appeared in jawed vertebrates. There is no strong support for any particular order of appearance of each KHDBRS subgroup, which rather suggests that triplication took place in a narrow time window after the split between hyperoartia and jawed vertebrates. Quaking/How form another monophyletic group of proteins present in early metazoans (Hydra and sea anemone). There is no Quaking homologue in the current assembly of the lamprey genome. Abbreviations for species names: Pmar (*Petromyzon marinus*); Hsap (*Homo sapiens*); Rrat (*Rattus rattus*); Drer (*Danio rerio*); Ggal (*Gallus gallus*); Cint (*Ciona intestinalis*); Spur (*strongylocentrotus purpuratus*); Skow (*Saccoglossus kowalevski*); Xtro (*Xenopus tropicalis*); Bflo (*Branchiostoma floridae*); Aaeg (*Aedes aegypti*); Dmel (*Drosophila melanogaster*); Odio (*Oikopleura dioica*); Cele (*C. elegans*); Hmag (*Hydra magnipapillata*); Nvec (*Nematostella vectensis*); Mbre (*Monosiga brevicollis*); Scer (*Saccharomyces cerevisiae*).
(PDF)

Figure S2 Percentage Splicing Inclusion values in for 792 ASEs in wild type (X axis) and knockout brain (Y axis) with strongly repressed exons arrowed.
(PDF)

Figure S3 T-STAR protein is expressed in regions CA1–CA3 of the mouse hippocampus but not the dentate gyrus. The annotations and scale bar are used as in Figure 4.
(PDF)

Table S1 Occurrence of UWAA motifs in the Neurexin genes. Both the complete *Neurexin1* and *Neurexin3* genes had an overall deficit for both TTAA and TTAATTAA sequences. The whole *Neurexin2* gene also had a slight TTAA deficit, but an excess of the extended TWAATWAA and TTAATTAA motifs. For the regions of the *Neurexin* genes which surround the AS4 alternative exon only (E−1 to E+1, which includes the upstream and downstream flanking regions), the *Neurexin2* gene had a significant excess of TWAA and TTAA motifs downstream of AS4, and a very strong excess of TWAATWAA and TTAATTAA. In contrast, the equivalent regions in *Neurexin1* showed a moderate deficit of TTAA and a random distribution in *Neurexin3*. z-score = (observed−expected)/√ (variance). Z-scores above 1.96 or below −1.96 reject the hypothesis H_0_ that word occurrence distribution is random at the 5% significance level.
(PDF)
