# Supplementary figures and images for: The Tissue-Specific RNA Binding Protein T-STAR Controls Regional Splicing Patterns of Neurexin Pre-mRNAs in the Brain
Source: PLoS Genet. 2013 Apr 25;9(4):e1003474. doi: 10.1371/journal.pgen.1003474 (PMC3636136; doi:10.1371/journal.pgen.1003474)

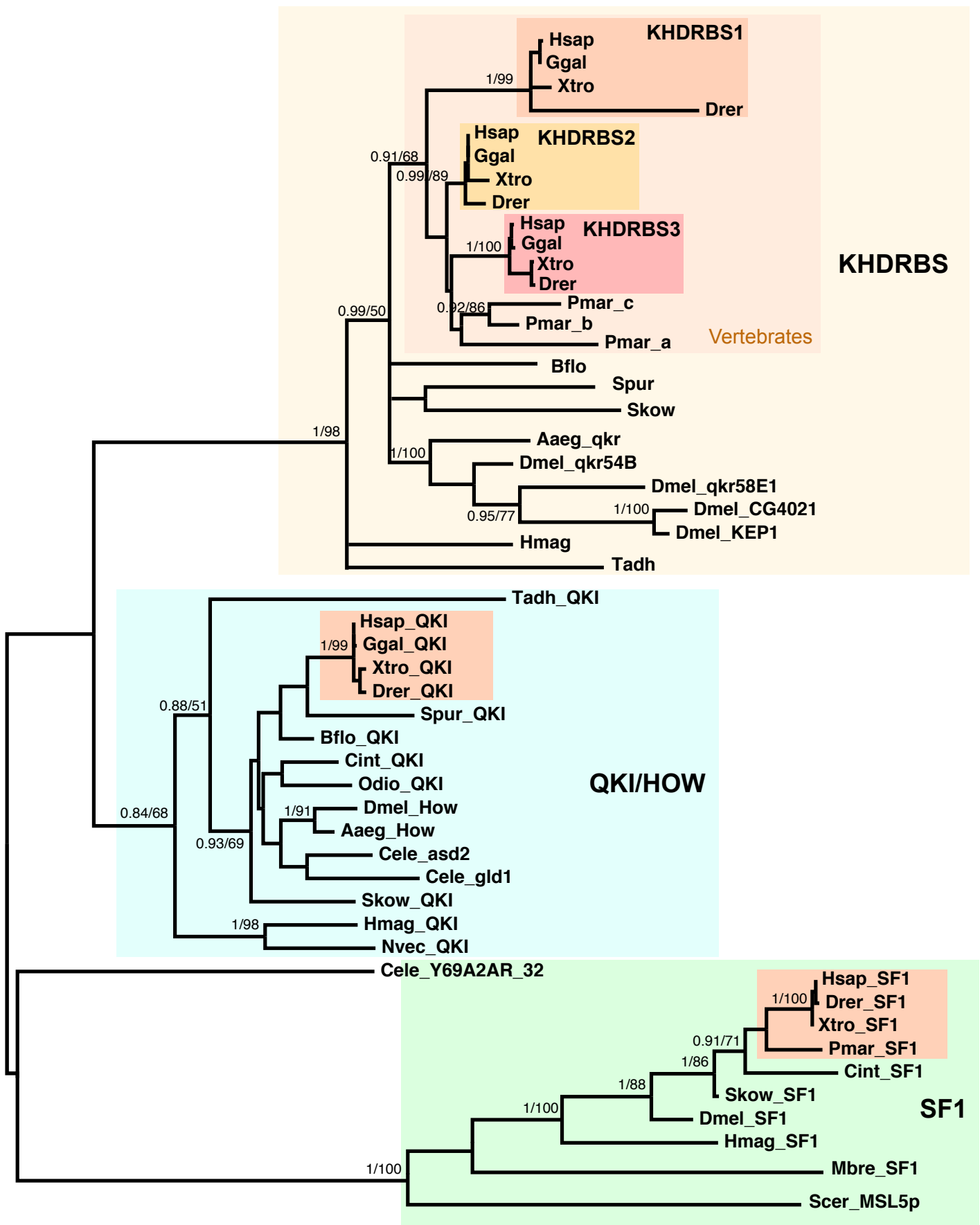

Figure S1 -Elliott

Supplement: Figure S1 — The Khdrbs3 gene evolved via gene triplication early in the radiation of bony vertebrates. Cladogram indicating the evolutionary profiles of the KHDRBS proteins, using the STAR protein SF1 as an external outgroup since SF1/MSL5p is the only GSG protein found in unicellular eukaryotes like S. cerevisiae. The KHDBRS proteins form a well supported monophyletic group, in which members are already present in early metazoans (Hydra, Trichoplax). Drosophila KEP1 and related proteins belong to this group. Whereas cephalochordates and insects code for KHSRBS-related sequences, no homolog was found in Ciona intestinalis nor in C. elegans, indicating a secondary loss of Sam68 genes in these taxons. Blast analysis of the lamprey genome suggests that it encodes only 4 STAR proteins. One is SF1, the other three derive from lamprey-specific duplications of a single KHDBRS gene. The three subgroups T-STAR, Sam68 and SLM-1 (KHDBRS1, 2 and 3 genes) appeared in jawed vertebrates. There is no strong support for any particular order of appearance of each KHDBRS subgroup, which rather suggests that triplication took place in a narrow time window after the split between hyperoartia and jawed vertebrates. Quaking/How form another monophyletic group of proteins present in early metazoans (Hydra and sea anemone). There is no Quaking homologue in the current assembly of the lamprey genome. Abbreviations for species names: Pmar (Petromyzon marinus); Hsap (Homo sapiens); Rrat (Rattus rattus); Drer (Danio rerio); Ggal (Gallus gallus); Cint (Ciona intestinalis); Spur (strongylocentrotus purpuratus); Skow (Saccoglossus kowalevski); Xtro (Xenopus tropicalis); Bflo (Branchiostoma floridae); Aaeg (Aedes aegypti); Dmel (Drosophila melanogaster); Odio (Oikopleura dioica); Cele (C. elegans); Hmag (Hydra magnipapillata); Nvec (Nematostella vectensis); Mbre (Monosiga brevicollis); Scer (Saccharomyces cerevisiae). (PDF) [file pgen.1003474.s003.pdf]

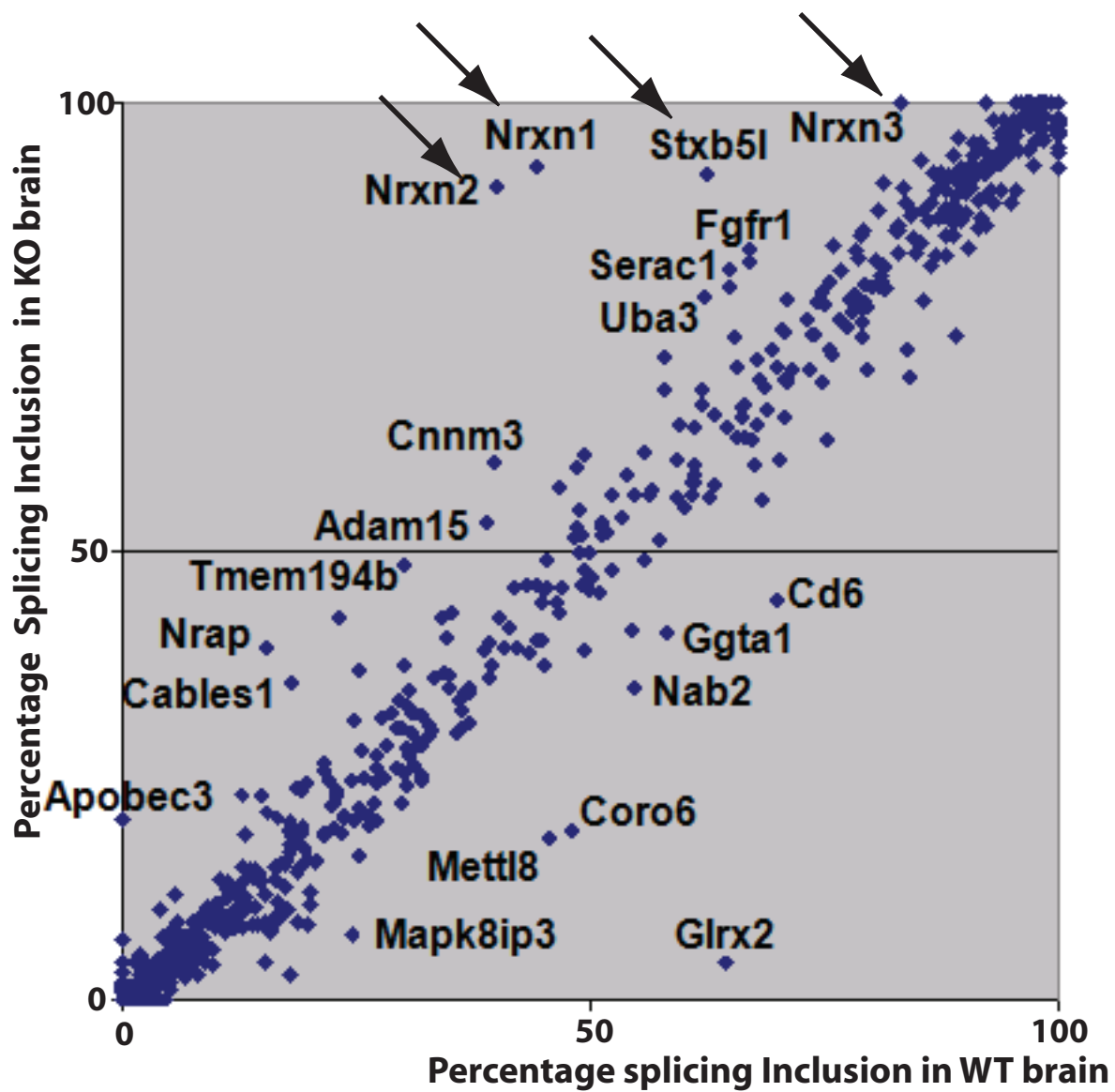

Supplement: Figure S2 — Percentage Splicing Inclusion values in for 792 ASEs in wild type (X axis) and knockout brain (Y axis) with strongly repressed exons arrowed. (PDF) [file pgen.1003474.s004.pdf]

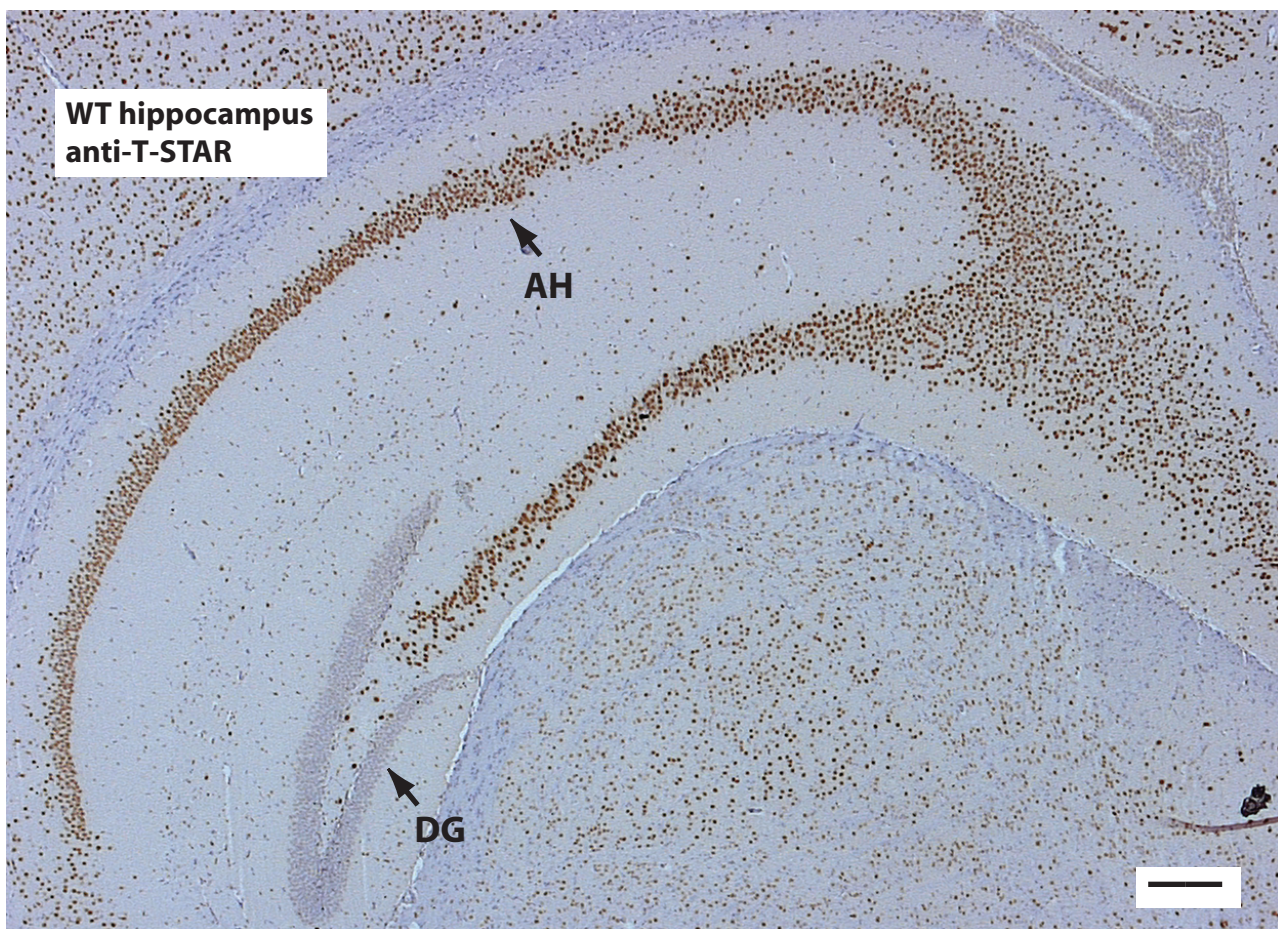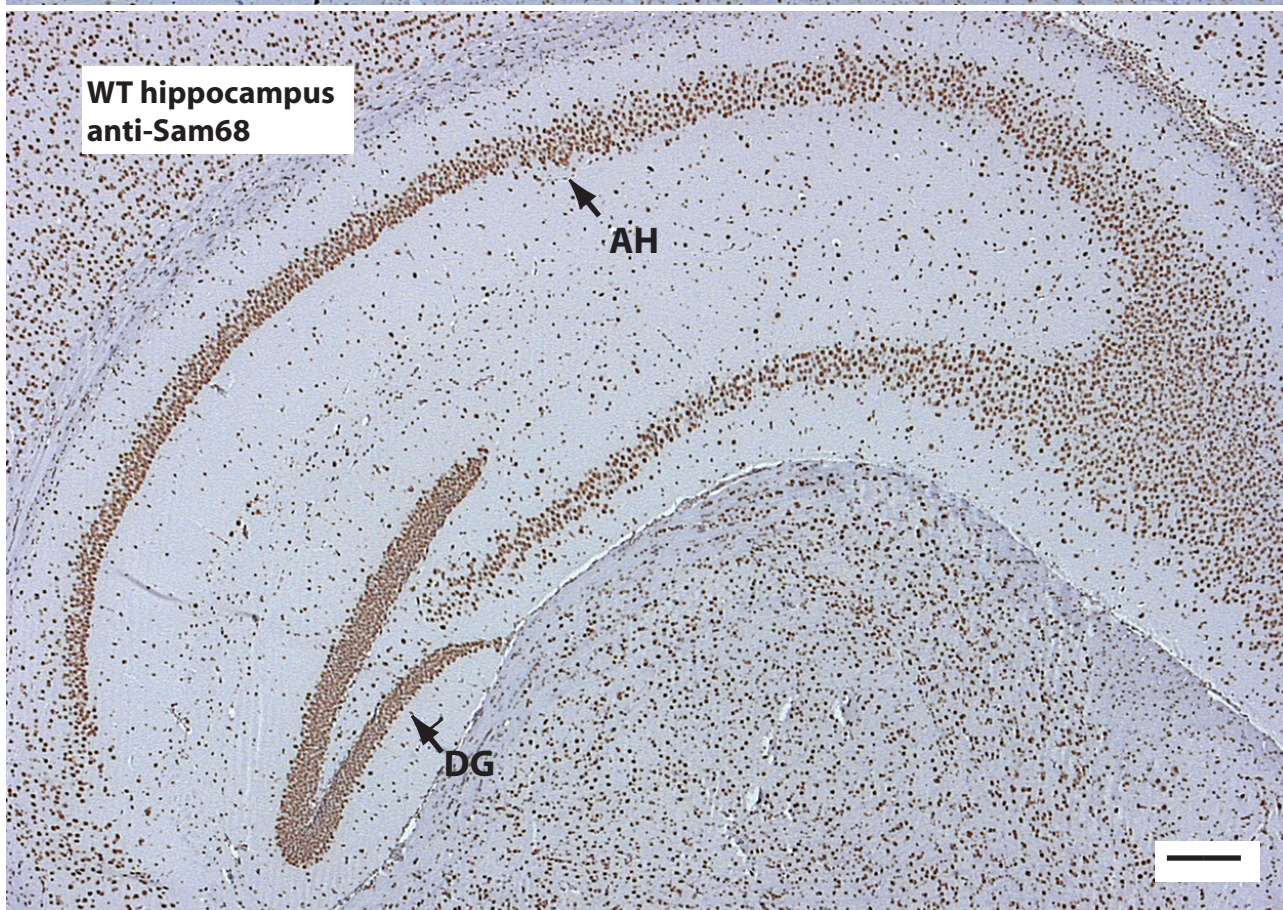

Supplement: Figure S3 — T-STAR protein is expressed in regions CA1–CA3 of the mouse hippocampus but not the dentate gyrus. The annotations and scale bar are used as in Figure 4. (PDF) [file pgen.1003474.s005.pdf]
