# Supplementary material for: The Tissue-Specific RNA Binding Protein T-STAR Controls Regional Splicing Patterns of Neurexin Pre-mRNAs in the Brain
Source: PLoS Genet. 2013 Apr 25;9(4):e1003474. doi: 10.1371/journal.pgen.1003474 (PMC3636136; doi:10.1371/journal.pgen.1003474)
Supplement: Table S1 — Occurrence of UWAA motifs in the Neurexin genes. Both the complete Neurexin1 and Neurexin3 genes had an overall deficit for both TTAA and TTAATTAA sequences. The whole Neurexin2 gene also had a slight TTAA deficit, but an excess of the extended TWAATWAA and TTAATTAA motifs. For the regions of the Neurexin genes which surround the AS4 alternative exon only (E−1 to E+1, which includes the upstream and downstream flanking regions), the Neurexin2 gene had a significant excess of TWAA and TTAA motifs downstream of AS4, and a very strong excess of TWAATWAA and TTAATTAA. In contrast, the equivalent regions in Neurexin1 showed a moderate deficit of TTAA and a random distribution in Neurexin3. z-score = (observed−expected)/√ (variance). Z-scores above 1.96 or below −1.96 reject the hypothesis H0 that word occurrence distribution is random at the 5% significance level. (PDF) [file pgen.1003474.s006.pdf]

| Full-length genes: | | | Observed | Expected | Variance | z-score |
|---|---|---|---|---|---|---|
| HsNRXN1 | TWAA | | 21852 | 21655.195 | 9780.823 | 1.99 |
| HsNRXN1 | TTAA | deficit | 10352 | 11799.685 | 9804.668 | **-14.6203** |
| HsNRXN1 | TWAATWAA | | 477 | 474.426 | 485.435 | 0.1168 |
| HsNRXN1 | TTAATTAA | deficit | 89 | 125.188 | 127.129 | **-3.2095** |
| HsNRXN2 | TWAA | | 481 | 532.419 | 575.734 | -2.143 |
| HsNRXN2 | TTAA | deficit | 231 | 291.576 | 270.996 | **-3.6798** |
| HsNRXN2 | TWAATWAA | excess | 9 | 2.608 | 2.63 | **3.9419** |
| HsNRXN2 | TTAATTAA | excess | 6 | 0.782 | 0.786 | **5.8863** |
| HsNRXN3 | TWAA | | 24573 | 24670.899 | 11713.26 | -0.9046 |
| HsNRXN3 | TTAA | deficit | 11333 | 13810.078 | 11768.11 | **-22.8342** |
| HsNRXN3 | TWAATWAA | | 530 | 541.366 | 550.429 | -0.4844 |
| HsNRXN3 | TTAATTAA | deficit | 103 | 123.753 | 38.36 | **-3.3507** |
| | | | | | | |
| From the (-1) exon to the (+1) exon | | | | | | |
| NRXN1r | TWAA | | 810 | 840.088 | 793.521 | -1.0681 |
| NRXN1r | TTAA | deficit | 369 | 419.722 | 347.302 | **-2.7217** |
| NRXN1r | TWAATWAA | | 10 | 18.463 | 18.907 | -1.9464 |
| NRXN1r | TTAATTAA | | 1 | 4.609 | 4.682 | -1.6678 |
| NRXN2r | TWAA | excess | 78 | 45.101 | 48.204 | **4.7385** |
| NRXN2r | TTAA | excess | 33 | 20.972 | 19.498 | **2.7239** |
| NRXN2r | TWAATWAA | excess | 5 | 0.26 | 0.262 | **9.2572** |
| NRXN2r | TTAATTAA | excess | 4 | 0.056 | 0.056 | **16.6034** |
| NRXN3r | TWAA | | 690 | 654.69 | 631.784 | 1.4048 |
| NRXN3r | TTAA | | 330 | 332.5 | 279.187 | -0.1496 |
| NRXN3r | TWAATWAA | | 14 | 12.547 | 12.828 | 0.4056 |
| NRXN3r | TTAATTAA | | 5 | 3.236 | 3.283 | 0.9733 |
